# Tests for segregation distortion in tetraploid F1 populations

**DOI:** 10.1007/s00122-025-04816-z

**Published:** 2025-01-16

**Authors:** David Gerard, Mira Thakkar, Luis Felipe V. Ferrão

**Affiliations:** 1 Department of Mathematics and Statistics, American University, Washington, DC 20016 USA; 2 Horticultural Sciences Department, University of Florida, Gainesville, FL 32611 USA

## Abstract

**Key message:**

In tetraploid F1 populations, traditional segregation distortion tests often inaccurately flag SNPs due to ignoring polyploid meiosis processes and genotype uncertainty. We develop tests that account for these factors.

**Abstract:**

Genotype data from tetraploid F1 populations are often collected in breeding programs for mapping and genomic selection purposes. A common quality control procedure in these groups is to compare empirical genotype frequencies against those predicted by Mendelian segregation, where SNPs detected to have *segregation distortion* are discarded. However, current tests for segregation distortion are insufficient in that they do not account for double reduction and preferential pairing, two meiotic processes in polyploids that naturally change gamete frequencies, leading these tests to detect segregation distortion too often. Current tests also do not account for genotype uncertainty, again leading these tests to detect segregation distortion too often. Here, we incorporate double reduction, preferential pairing, and genotype uncertainty in likelihood ratio and Bayesian tests for segregation distortion. Our methods are implemented in a user-friendly R package, menbayes. We demonstrate the superiority of our methods to those currently used in the literature on both simulations and real data.

**Supplementary Information:**

The online version contains supplementary material available at 10.1007/s00122-025-04816-z.

## Introduction

Polyploids, organisms containing more than two sets of chromosomes, play a dominant role in many sectors of agriculture (Udall and Wendel 2006). Consequently, numerous breeding programs are dedicated to the agricultural improvement of polyploids (Ferrão et al. 2018; Shirasawa et al. 2017; Amadeu et al. 2021; Lau et al. 2022). In these programs, breeders frequently generate “F1 populations” of full siblings for various tasks, such as QTL mapping (Amadeu et al. 2021), linkage mapping (Bourke et al. 2018; Mollinari and Garcia 2019), and genomic selection (Ferrão et al. 2021), all of which are crucial for crop improvement.

In these F1 populations, offspring genotypes should roughly adhere to the laws of Mendelian segregation (Mendel 1866). Hence, it is customary to use a chi-squared test to compare observed offspring genotype frequencies with those predicted by Mendelian segregation to identify problematic SNPs caused, for example, by sequencing errors, mapping biases, or amplification biases (Bourke et al. 2015; Cappai et al. 2020; Mollinari et al. 2020; Batista et al. 2021, e.g.). Such deviations are referred to as *segregation distortion*. However, there are two significant limitations to using the chi-squared test in these scenarios. First, many polyploids naturally undergo double reduction and (partial) preferential pairing (Voorrips and Maliepaard 2012), two meiotic processes that can lead to deviations from classical gamete frequencies even for well-behaved SNPs. The resulting offspring genotype frequencies heavily depend on the type of polyploid (allo, auto, or segmental) (Doyle and Egan 2010), necessitating tests for F1 populations that can adapt to these varying types. Second, the chi-squared test does not account for genotype uncertainty, a major concern in polyploid genetics (Gerard et al. 2018; Gerard and Ferrão 2019) that can adversely impact many genomics methods.

In this paper, we develop a model for the genotype frequencies of a biallelic locus in an F1 tetraploid population that allows for arbitrary levels of double reduction and preferential pairing (Section Generalized gamete frequencies). This fills a gap in the literature, as most approaches only account for either double reduction or preferential pairing, but not both (Appendix S1). We harness this new model to develop likelihood ratio tests (LRTs) for segregation distortion, optionally accounting for genotype uncertainty through genotype likelihoods (Li 2011) (Section Likelihood ratio tests for segregation distortion). To take advantage of the benefits of a Bayesian paradigm approach, we further develop Bayesian tests for segregation distortion (Section Bayesian tests for segregation distortion). We demonstrate our methods both on simulations (Sections Null simulations and Alternative simulations) and on a dataset of tetraploid blueberries (Section Blueberries).

### Related work

In the context of modeling preferential pairing and double reduction, previous studies have primarily focused on estimation rather than testing. A comprehensive review of these studies is provided in Appendix S1. In this section, we focus on the related work that emphasizes testing.

Tests have been created to evaluate the related hypothesis of random mating. A likelihood ratio test for random mating was created in Appendix C of Gerard (2022), exact tests were explored in Matoka Nana (2023), and Bayesian tests were developed in Gerard (2023). Many of the approaches in those papers account for genotype uncertainty. Random mating is applicable to S1 populations (a generation of selfing) as all individuals have their gametes drawn from the same distribution and are randomly selected during fertilization. However, F1 populations violate the random mating hypothesis at loci where parental genotypes differ since the gametes from each parent are drawn from different distributions. Thus, these tests are not generally applicable in our scenario of F1 populations.

The work most closely related to ours, particularly in terms of testing, is likely the tests implemented by the polymapR software (Bourke et al. 2018). This software offers tests for segregation distortion in tetraploids within its function checkF1(). The process involves analyzing each segregation pattern, which can be (i) polysomic in both parents, (ii) disomic in both parents, or (iii) polysomic in one and disomic in the other, followed by conducting a chi-squared test based on that specific segregation pattern. This test is performed using only the possible genotypes. For instance, if the potential offspring genotypes from parent genotypes are 0 and 1, but some offspring genotypes of 2 are observed, these genotypes are excluded from the chi-squared test. A separate one-sided binomial test is conducted for “invalid” genotypes (considering the parental genotypes and their segregation patterns), with an expected proportion of invalid genotypes hard-coded at less than 3%. The product of the *p*-values from both the chi-squared and binomial tests is then calculated, and the maximum of these is used as the indicator of segregation distortion. This method resembles a minimum chi-squared test (Berkson 1980) where the authors explore the discrete parameter space of fully disomic and fully polysomic parents, albeit using a somewhat ad-hoc criterion. Our approach, in contrast, is more principled and allows for a full exploration of the parameter space of gamete frequencies resulting from both double reduction and partial preferential pairing, rather than limiting to completely polysomic or completely disomic inheritance.

Bourke et al. (2018) also account for genotype uncertainty by using posterior probabilities as inputs but do so in an ad-hoc way. They sum the posterior probability of each genotype over the individuals to get a total count for each genotype, they then round counts below some preset threshold down to zero, and renormalize the resulting count vector to sum to the sample size of the offspring. They then apply the same approach as in the known genotype case to this estimated vector of counts.

We will show in the Section Null simulations that our approach has advantages to that of Bourke et al. (2018).

## Materials and methods

### Generalized gamete frequencies

We begin by describing the hypothesis of no segregation distortion. We assume that we are working with a single biallelic locus, and we are concerned with the genotype frequencies of an F1 population of polyploids at this locus. Though our manuscript focuses on tetraploids, we will write out equations for an arbitrary (even) ploidy level, *K*, when they are appropriate and correct for arbitrary ploidies. If desired, one can set $K=4$ throughout the following. Let $\varvec{q} = (q_0,q_1,\ldots ,q_K)$ be the genotype frequencies of a *K*-ploid F1 population, where $q_k$ is the proportion of offspring expected to have genotype *k*. Each parent provides a gamete to each offspring, and the “gamete frequencies” of parent $j \in \{1, 2\}$ will be denoted by $\varvec{p}_j = (p_{j0}, p_{j1}, \ldots , p_{j,K/2})$. That is, $p_{jk}$ is the proportion of parent *j*’s gametes expected to have genotype *k*. Because each offspring genotype is the sum of the two (independent) parental gamete genotypes, we can write $\varvec{q}$ as a discrete linear convolution of $\varvec{p}_1$ and $\varvec{p}_2$,

1 $$\begin{aligned} q_k = \sum _{i = \max (0, k - K/2)}^{\min (k,K/2)} p_{1i}p_{2,k-i}. \end{aligned}$$

In tetraploids, the subject of our paper, this corresponds to

2 $$\begin{aligned} \begin{aligned} q_0&= p_{10}p_{20},\\ q_1&= p_{10}p_{21} + p_{11}p_{20},\\ q_2&= p_{10}p_{22} + p_{11}p_{21} + p_{12}p_{20},\\ q_3&= p_{11}p_{22} + p_{12}p_{21}, \text { and}\\ q_4&= p_{12}p_{22}. \end{aligned} \end{aligned}$$

Not all values of $\varvec{p}_j$ are possible, and models for segregation correspond to models for the $\varvec{p}_j$’s based on each parental genotype. Let $\ell _j \in \{0,1,\ldots ,K\}$ be the genotype for parent *j*. For true autopolyploids that exhibit strict bivalent pairing, the $p_{jk}$’s are hypergeometric probabilities (Muller 1914; Serang et al. 2012),

3 $$\begin{aligned} p_{jk} = \frac{\left( {\begin{array}{c}\ell _j\\ k\end{array}}\right) \left( {\begin{array}{c}K - \ell _j\\ K/2 - k\end{array}}\right) }{\left( {\begin{array}{c}K\\ K/2\end{array}}\right) }. \end{aligned}$$

However, polyploids often exhibit some quadrivalent pairing, which can lead to the meiotic process of “double reduction”, the co-migration of sister chromatids segments into the same gamete (Mather 1935; Stift et al. 2010). Double reduction alters the gamete frequencies for polyploids. The characterization of these gamete frequencies was described in Fisher and Mather (1943) for autotetraploids and autohexaploids, before being generalized to arbitrary ploidy levels in Huang et al. (2019).

Additionally, many polyploids exhibit partial (or full) preferential pairing, where homologues preferentially (or exclusively) form bivalents during meiosis. Those that exhibit full disomic inheritance are called “allopolyploids” (Doyle and Egan 2010; Parisod et al. 2010), while those that exhibit partial preferential pairing are called “segmental allopolyploids” (Stebbins 1947) among other terms (Bourke et al. 2017). No model yet exists to incorporate both double reduction and preferential pairing at biallelic loci, though Stift et al. (2008) produced a model that incorporates both of these processes in tetraploids when each chromosome is distinguishable.

**Table 1:** At a single locus for a tetraploid, the distribution of the number, *x*, of alternative alleles sent to an offspring by a parent with dosage $\ell =$ 0, 1, 2, 3, or 4

	$x = 0$	$x = 1$	$x = 2$
$Pr(x|\ell =0)$	1	0	0
$Pr(x|\ell = 1)$	$\frac{1}{2} + \frac{1}{4}\beta \tau$	$\frac{1}{2} - \frac{1}{2}\beta \tau$	$\frac{1}{4}\beta \tau$
$Pr(x|\ell = 2)$	$\frac{1}{3}\beta \tau + \frac{1}{6}\tau + \frac{1}{4}(1-\tau )(1-\gamma )$	$-\frac{2}{3}\beta \tau + \frac{2}{3}\tau + \frac{1}{2}(1-\tau )(1 + \gamma )$	$\frac{1}{3}\beta \tau + \frac{1}{6}\tau + \frac{1}{4}(1-\tau )(1-\gamma )$
$Pr(x|\ell = 3)$	$\frac{1}{4}\beta \tau$	$\frac{1}{2} - \frac{1}{2}\beta \tau$	$\frac{1}{2} + \frac{1}{4}\beta \tau$
$Pr(x|\ell = 4)$	0	0	1

The probability of quadrivalent formation is $\tau$, $\beta$ is the probability of double reduction given quadrivalent formation, and $\gamma$ is the probability that chromosome pairing occurs along shared alleles given bivalent formation

**Table 2:** At a single locus for a tetraploid, the distribution of the number, *x*, of alternative alleles sent to an offspring by a parent with dosage $\ell =$ 0, 1, 2, 3, or 4

	$x = 0$	$x = 1$	$x = 2$
$Pr(x|\ell = 0)$	1	0	0
$Pr(x|\ell = 1)$	$\frac{1}{2} + \frac{1}{4}\alpha$	$\frac{1}{2} - \frac{1}{2}\alpha$	$\frac{1}{4}\alpha$
$Pr(x|\ell = 2)$	$\frac{1}{2}\alpha + \frac{1}{4}(1 - \alpha )(1 - \xi )$	$\frac{1}{2}(1 - \alpha )(1 + \xi )$	$\frac{1}{2}\alpha + \frac{1}{4}(1 - \alpha )(1 - \xi )$
$Pr(x|\ell = 3)$	$\frac{1}{4}\alpha$	$\frac{1}{2} - \frac{1}{2}\alpha$	$\frac{1}{2} + \frac{1}{4}\alpha$
$Pr(x|\ell = 4)$	0	0	1

The double reduction rate is $\alpha$ and the preferential pairing parameter is $\xi$. No preferential pairing corresponds to $\xi = 1/3$

For one of our contributions, in Appendix S2, we developed a model that incorporates both double reduction and preferential pairing in the gamete frequencies of tetraploids. These frequencies are tabulated in Table 1 in terms of three parameters: the probability of quadrivalent formation, $\tau$, the probability of double reduction given quadrivalent formation, $\beta$, and the probability that chromosomes with the same alleles will pair given bivalent formation, $\gamma$. We further show that this three-parameter model can be reduced to a model with two parameters (Table 2): the double reduction rate, $\alpha$, and the preferential pairing parameter, $\xi$, where a value of $\xi = 1/3$ indicates strict polysomic inheritance and values of $\xi = 0$ or 1 indicate strict disomic inheritance. Our model is nicely connected with others in the literature. Our model is derived from that of Stift et al. (2008), reduced to biallelic loci, when the parameters of that model are reinterpreted. Furthermore, when $\xi = 1/3$ this model reduces to that of Fisher and Mather (1943) (Appendix S3).

The benefit of our model is that it can account for a wider range of possible gamete frequencies than models that incorporate double reduction alone. That is, some well-behaved SNPs (with some amount of preferential pairing) cannot have their genotype frequencies modeled appropriately with double reduction alone. To see this, consider that for $\ell = 2$, $p_0 = p_2$, we can order the gamete frequencies by their value of $p_1 = 1 - p_0 - p_2$. When accounting for double reduction alone, the range of gamete frequencies when $\ell = 2$ goes from $\varvec{p} = (2, 5, 2) / 9 \approx (0.22, 0.56, 0.22)$ (for $\alpha = 1/6$) to $\varvec{p} = (1, 4, 1) / 6 \approx (0.17, 0.67, 0.17)$ (for $\alpha = 0$). When accounting for both double reduction and preferential pairing, the range of gamete frequencies goes from $\varvec{p} = (1, 2, 1) / 4$ (for $\xi = 0$ and $\alpha = 0$) to $\varvec{p} = (0, 1, 0)$ (for $\xi = 1$ and $\alpha = 0$). Thus, values of $1/2< p_1 < 5/9$ and $2/3 < p_1 \le 1$ cannot be accounted for by double reduction alone but can be accounted for when including preferential pairing.

**Fig. 1:**
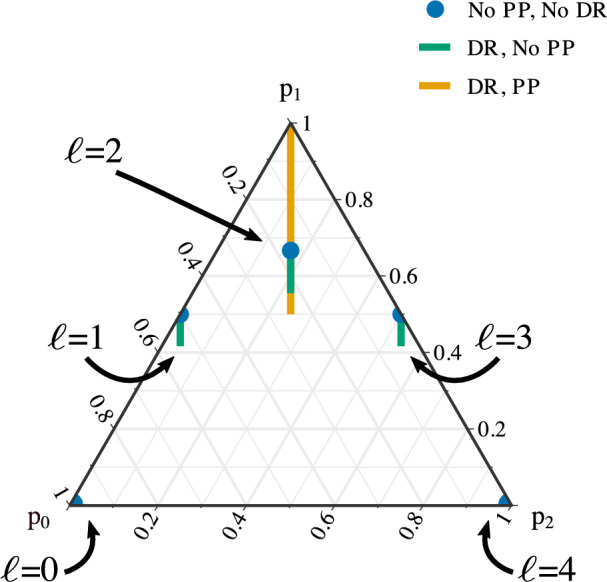
Ternary plot (Hamilton and Ferry 2018) of the gamete frequencies $(p_0,p_1,p_2)$ of a tetraploid under different hypotheses of meiosis. The parent’s genotype is denoted by $\ell \in \{0,1,2,3,4\}$. The blue dots are the gamete frequencies of a true autotetraploid with no double reduction and no preferential pairing ($\alpha = 0$ and $\xi = 1/3$). The green lines are the gamete frequencies of a true autotetraploid with possible double reduction up to the maximum under the complete equational segregation model (Huang et al. 2019) ($\alpha \in [0,1/6]$ and $\xi = 1/3$). The orange line contains the gamete frequencies, when $\ell = 2$, under arbitrary levels of double reduction and preferential pairing ($\alpha \in [0,1/6]$ and $\xi \in [0,1]$)

In Fig. 1, we graphically represent the gamete frequencies under these different models for meiosis via a ternary plot (Hamilton and Ferry 2018). There, we see that a model that does not account for double reduction and preferential pairing only allows for gamete frequencies at the blue dots, while a model that accounts for double reduction and not preferential pairing only allows for gamete frequencies at the blue dots and green lines. Our new model that allows for both double reduction and preferential pairing allows for gamete frequencies at the blue dots, and green and orange lines, which is a much larger possible space of gamete frequencies.

In Sections Likelihood ratio tests for segregation distortion and Bayesian tests for segregation distortion, we will use our new model to construct tests for segregation distortion in F1 populations of tetraploids. There, we will assume that the two parents share a common double reduction rate ($\alpha$), but each has their own preferential pairing parameter ($\xi _1$ and $\xi _2$). It would be incorrect to fix $\xi _1$ to equal $\xi _2$ due to the interpretation of this parameter in term of pairing frequencies based on allele compositions (see Section Discussion).

Concerning our new model for gamete frequencies, unfortunately neither the two-parameter nor the three-parameter model are identified when the double reduction rate and the preferential pairing parameter are together. That is, the models are not identified when a parent is duplex ($\ell = 2$). One can see this, for example, by noting that, when $\ell = 2$, $\alpha = 1/6$ and $\xi = 1/3$ results in the same gamete frequencies as $\alpha = 0$ and $\xi = 1/9$. Since $\xi$ only appears in duplex parents (Table 2), this means that one cannot estimate $\xi$ (or $\alpha$ when $\ell = 2$) using just a single biallelic locus (without further assumptions). However, our model indicates that one need not worry about preferential pairing at loci where $\ell = 1$ or 3 and can, conceivably, use these loci to estimate the double reduction rate, $\alpha$. However, we will see that such estimates, using just a single biallelic locus, are biased and highly variable (Section Null simulations). We note that though the model is unidentified when $\ell = 2$, this is not a major issue for our purpose of hypothesis testing. The unidentifiability affects the number of degrees of freedom calculation for the LRTs of Section Likelihood ratio tests for segregation distortion and merely affects the prior distribution over the null parameter space for our Bayesian tests in Section Bayesian tests for segregation distortion.

### Likelihood ratio tests for segregation distortion

Our goal in this section is to construct LRTs to compare the following two hypotheses.

- $H_0$: $\varvec{p}_j$ is defined by Table 2 via parameters $\alpha$ and $\xi _j$, and $\varvec{q}$ is defined by (2).
- $$H_A$$: Not $$H_0$$

We can graphically represent these two hypotheses via the ternary plot (Hamilton and Ferry 2018) in Fig. 1. The null hypothesis is that the gamete frequencies lie on the blue dots or the green or orange lines. One possible scenario of the alternative hypothesis is that the gamete frequencies lie anywhere else on the 2-simplex. More generally, the alternative hypothesis states that the genotype frequencies are anywhere on the 4-simplex that are not consistent with F1 genotype frequencies under double reduction and preferential pairing.

When considering $H_0$, we will denote the functional dependence of $\varvec{q}$ on $\alpha$, $\xi _1$, $\xi _2$, $\ell _1$, and $\ell _2$ by $\varvec{q}(\alpha , \xi _1, \xi _2, \ell _1, \ell _2)$ if using the two-parameter model (Table 2). If using the three-parameter model (Table 1), we will denote this dependence by $\varvec{q}(\tau , \beta , \gamma _1, \gamma _2, \ell _1, \ell _2)$. We construct these tests in three scenarios: one where the genotypes are known, one where parental genotypes are known but offspring genotype uncertainty is represented through genotype likelihoods (Li 2011), and one where all individuals have genotype uncertainty represented through genotype likelihoods.

We begin with the case when the genotypes are known. Let $x_k$ be the number of individuals with genotype $k \in \{0,1,\ldots ,K\}$, which we collect into the vector $\varvec{x} = (x_0, x_1, \ldots , x_K)$. We denote the sample size by $n = \sum _{k=0}^Kx_k$. Then, given genotype frequencies $\varvec{q}$, we have that $\varvec{x}$ follows a multinomial distribution,

4 $$\begin{aligned} f(\varvec{x}|\varvec{q}) = \frac{n!}{x_0!\cdots x_K!}q_0^{x_0}\cdots q_K^{x_K}. \end{aligned}$$

The maximum likelihood estimate of $\varvec{q}$ under the alternative is $\hat{\varvec{q}}_A = \varvec{x} / n$. We maximize the likelihood function, $f(\varvec{x}|\varvec{q}(\tau , \beta , \gamma _1, \gamma _2, \ell _1, \ell _2))$, over $0 \le \tau , \gamma _1, \gamma _2 \le 1$ and $0 \le \beta \le c$, where *c* is the maximum rate of double reduction. By default, we set $c = 1/6$, the maximum under the complete equational segregation model (Mather 1935). We do this maximization using gradient ascent (Byrd et al. 1995) to obtain $\hat{\varvec{q}}_0$. We then obtain the likelihood ratio statistic

5 $$\begin{aligned} \lambda = -2 (\log f(\varvec{x}|\hat{\varvec{q}}_0) - \log f(\varvec{x}|\hat{\varvec{q}}_A)), \end{aligned}$$

and compare $\lambda$ to an appropriate $\chi ^{2}$ distribution to obtain a *p*-value.

Calculating the null distribution of this test is rather difficult, as the parameters under the null might lie on or near the boundary of the parameter space, which requires special considerations (Self and Liang 1987; Mitchell et al. 2019; Leung and Sturma 2024). Thus, we applied the data-dependent degrees of freedom strategy of Susko (2013), which we describe now. The number of parameters under the null is equivalent to the dimension of the null parameter space, which can be visualized in Fig. 1. If $\ell _1,\ell _2 \in \{0, 4\}$, then the number of parameters under the null is 0, because the parameter space is 0 dimensional (a single dot) in Fig. 1. If $\ell \in \{1,2,3\}$, then the number of parameters under the null is 1 if the parameters are estimated in the interior of the parameter space, because the parameter space is 1 dimensional (a single line) in Fig. 1. The number of parameters under the null is 0 if they are estimated on the boundary of the parameter space (at the ends of the lines in Fig. 1). To calculate the number of parameters under the alternative, we note that, if the null were true, some offspring genotypes would be impossible. The test returns a *p*-value of 0 if any of these “impossible” genotypes are observed; otherwise, the number of parameters under the alternative is the number of theoretically possible genotypes minus 1. The number of degrees of freedom for the chi-squared distribution is the difference between the number of parameters under the alternative and under the null. This strategy is guaranteed to asymptotically control type I error but might be asymptotically conservative (Susko 2013).

We now consider the LRT when parental genotypes are known, but offspring use genotype likelihoods (Li 2011). Let $g_{ik}$ be the genotype likelihood for individual $i = 1,2,\ldots ,n$ for genotype $k = 0,1,\ldots ,K$. That is, $g_{ik}$ is the probability of the data (sequencing, microarray, or otherwise) for individual *i* given that the genotype for that individual is *k*. Then, given these genotype likelihoods, we have the likelihood for these data is

6 $$\begin{aligned} f(\varvec{G}|\varvec{q}) = \prod _{i=1}^n\sum _{k=0}^Kg_{ik}q_{k}. \end{aligned}$$

The maximum likelihood estimate of $\varvec{q}$ under the alternative can be found by the EM algorithm of Li (2011), which we denote by $\hat{\varvec{q}}_A$. We maximize $f(\varvec{G}|\varvec{q}(\tau , \beta , \gamma _1, \gamma _2, \ell _1, \ell _2))$ over $0 \le \tau , \gamma _1, \gamma _2 \le 1$ and $0 \le \beta \le c$, using gradient ascent (Byrd et al. 1995), to obtain $\hat{\varvec{q}}_0$. We obtain a *p*-value by comparing the likelihood ratio statistic,

7 $$\begin{aligned} \lambda = -2 (\log f(\varvec{G}|\hat{\varvec{q}}_0) - \log f(\varvec{G}|\hat{\varvec{q}}_A)), \end{aligned}$$

to an appropriate $\chi ^{2}$ distribution to obtain a *p*-value.

To obtain the number of degrees of freedom of this test, we again take the approach of Susko (2013). The number of parameters under the null is the same as in the known genotype case. The number of parameters under the alternative is 4 minus the number of the $q_k$’s that are both theoretically 0 under the null and are estimated to be 0 under the alternative. The number of degrees of freedom of the test is the difference between the number of parameters under the alternative and the null. Again, this strategy is guaranteed to asymptotically control from type I error but might be asymptotically conservative (Susko 2013).

We now consider the case when both parents and offspring use genotype likelihoods. Let $\varvec{a} = (a_0, a_1, \ldots , a_K)$ be the genotype likelihoods for parent 1, and let $\varvec{b} = (b_0, b_1, \ldots , b_K)$ be the genotype likelihoods for parent 2. We perform the LRT by first maximizing the following likelihood over the parent genotypes

8 $$\begin{aligned} a_{\ell _1}b_{\ell _2}f(\varvec{G}|\varvec{q}(\tau , \beta , \gamma _1, \gamma _2, \ell _1, \ell _2)). \end{aligned}$$

We then run the LRT as if the estimated parent genotypes were the known true parent genotypes.

### Bayesian tests for segregation distortion

To take advantage of the many benefits of Bayesian analysis, we developed Bayesian tests for segregation distortion. In particular to our case, Bayesian tests can more easily adapt to non-identifiable models, as this just alters the prior distribution over a parameter space. But, there are other advantages, such as ease of interpretability and consistency under the null (O’Hagan 1994, Section 7.52). The Bayesian testing paradigm consists of calculating a Bayes factor (BF) defined as the ratio of marginal likelihoods under the two hypotheses:

9 $$\begin{aligned} \textrm{BF}&= \frac{\textrm{Pr}(\text {data}|H_0)}{\textrm{Pr}(\text {data}|H_1)}\nonumber \\&= \frac{\int f(\text {data}|\varvec{q}(\tau , \beta , \gamma _1, \gamma _2, \ell _1, \ell _2))\pi _0(\tau ,\beta ,\gamma _1,\gamma _2,\ell _1,\ell _2) \textrm{d}\tau \textrm{d}\beta \textrm{d}\gamma _1\textrm{d}\gamma _2}{\int f(\text {data}|\varvec{q})\pi _1(\varvec{q}) \textrm{d}\varvec{q}}, \end{aligned}$$

where $\pi _0(\cdot )$ is the prior under the null, $\pi _1(\cdot )$ is the prior under the alternative, and $f(\text {data}|\varvec{q})$ is one of the likelihoods we consider, either equation (4) or (6). When parent genotypes are not known, we estimate the parent genotypes using maximum likelihood, as in Section Likelihood ratio tests for segregation distortion, and use likelihood (6) as if the parent genotypes were known.

For the null, we need to specify priors over $\tau$, the probability of quadrivalent formation, $\beta$, the probability of double reduction given quadrivalent formation, and $\gamma _j$, the probability of a AA:aa pairing given bivalent formation in parent $j = 1,2$. Our default selection is as follows,

10 $$\begin{aligned} \tau&\sim {{\,\textrm{Unif}\,}}(0, 1), \end{aligned}$$

11 $$\begin{aligned} \beta&\sim {{\,\textrm{Unif}\,}}(0, 1/6), \text { and} \end{aligned}$$

12 $$\begin{aligned} \gamma _1,\gamma _2&\sim {{\,\textrm{Beta}\,}}(5/9, 10/9). \end{aligned}$$

The upper bound on $\beta$ was chosen based on the maximum rate of double reduction, provided by the complete equational segregation model of meiosis (Mather 1935; Huang et al. 2019). The prior on the $\gamma _j$’s was created so that the mean would be 1/3, the value under tetrasomic inheritance (Appendix S3), and so that it would have the same variance as a uniform prior.

Under the alternative, we set the default prior for $\varvec{q}$ to be Dirichlet with concentration parameters $\varvec{1}_5/2 = (1,1,1,1,1)/2$. This was chosen based on empirical performance of the simulations in Section Results. A “natural” prior for $\varvec{q}$ might seem to be a uniform distribution over the 4-simplex, which would correspond to a Dirichlet distribution with concentration parameters $\varvec{1}_5$. However, Bayesian priors for proportions often use concentration parameters less than 1, e.g., in the context of Hardy-Weinberg testing (Bernardo and Tomazella 2010; Puig et al. 2017). This is also the Jeffreys prior for the multinomial distribution (Tuyl 2017), and so has theoretical justification as being, in a certain sense, uninformative.

All of these priors are adjustable by the user if they have additional prior knowledge on the meiotic process they study. E.g., if it is known that only some preferential pairing occurs, then the user could adjust the priors over $\gamma _1$ and $\gamma _2$ to be more concentrated around 1/3. In Appendix S4, we also demonstrate that our methods are relatively robust to prior selection.

Under the alternative, when genotypes are known, the marginal likelihood is the Dirichlet-multinomial (Mosimann 1962), which can be easily calculated. For all other models and likelihoods, we have to resort to simulation to estimate the marginal likelihoods. We implemented these models, using all three likelihoods and both the null and alternative priors, in Stan (Stan Development Team 2024a, b). We estimated marginal likelihoods (and therefore Bayes factors) via bridge sampling (Meng and Wong 1996; Gronau et al. 2020).

## Results

### Null simulations

To evaluate our methods, we ran simulations when the null of no segregation distortion was true. We varied the following parameters:

- The parent genotypes, $(\ell _1,\ell _2) \in \{(0,1),(0,2),(1,1),(1,2),(2,2)\}$.
- The sample size, $n \in \{20, 200\}$.
- The double reduction rate, $\alpha \in \{0,1/12,1/6\}$.
- The preferential pairing parameters, $\xi _1,\xi _2 \in \left\{ \frac{5}{3}\frac{\alpha }{1-\alpha }, \frac{1}{3}, 1 - \frac{10}{3}\frac{\alpha }{1-\alpha }\right\}$. We only varied the preferential pairing parameter $\xi _j$ when $\ell _j = 2$. When $\alpha = 1/6$, the bounds on the preferential pairing parameter constrains $\xi _1 = \xi _2 = 1/3$ (Theorem S2).
- The read-depth, $\{10, \infty \}$, where a read-depth of $\infty$ corresponds to the known genotype case.

Each replication, we simulated offspring genotypes using the model of Table 2. When genotypes were not known (a read-depth of 10), we further simulated offspring read-counts using the model of Gerard et al. (2018) under no allele bias, an overdispersion level of 0.01, and a sequencing error rate of 0.01. We then used the method of Gerard et al. (2018) to estimate offspring genotypes and obtain genotype likelihoods. Each replication, we fit the standard chi-squared test for segregation distortion (which compares the observed offspring genotypes against the theoretical genotype frequencies under no double reduction and no preferential pairing, (Muller 1914)), the polymapR test from Section Related work (Bourke et al. 2018), our new LRT from Section Likelihood ratio tests for segregation distortion, and our new Bayesian test from Section Bayesian tests for segregation distortion. For each unique combination of parameter values, we ran 200 replications.

Quantile-quantile plots against the uniform distribution of the *p*-values from the LRT of Section Likelihood ratio tests for segregation distortion, the standard chi-squared test, and the polymapR test of Section Related work (Bourke et al. 2018) are presented in Figures S1–S6. Since the null is true, the *p*-values should lie at or above the $y=x$ line to control type I error. Our new LRT is able to control type I error in all scenarios, often being unbiased and only sometimes being conservative (Figures S1–S2). In contrast, the chi-squared test does not control type I error when there is any double reduction or preferential pairing and fails to control type I error in almost all scenarios where there is genotype uncertainty (Figures S3–S4). The polymapR test fails to control type I error in some scenarios when genotypes are known, particularly when there is preferential pairing (Figure S5). When genotypes are not known, the polymapR test appears to control for type I error at small samples sizes for many scenarios (likely due to low power) but fails to control for type I error in most scenarios at larger sample sizes (Figure S6).

Box plots of the log Bayes factors from the Bayesian test of Section Bayesian tests for segregation distortion are presented in Figures S7–S8. Since the null is true, the log Bayes factors should be mostly positive, which is what we see. The only exception to this is in the case of true allopolyploids where the offspring exhibit “fixed heterozygosity” (Cornille et al. 2016), where the log Bayes factors are generally negative. This is likely because of the influence of our prior selection, which is not very informative toward allopolyploidy. Indeed, it is only under this scenario that prior specification appears to be vital (Appendix S4, Figures S9–S12), where priors that are informative toward allopolyploidy perform better. Though, in an applied setting, a researcher is likely aware that their organism might be a true allopolyploid, in which case they should use priors that are highly informative for allopolyploidy. One benefit of a Bayesian approach is that researchers can tailor their analyses based on their prior knowledge.

Our methods return estimates of the double reduction rate and preferential pairing parameters. However, when one of the parents is duplex, the double reduction rate and preferential pairing parameters are not identified (Section Generalized gamete frequencies). Since the preferential pairing parameter only appears when a parent is duplex (Table 2), this means that it is impossible to estimate the preferential pairing parameter using just a single biallelic locus (without further assumptions). It is conceivably possible to estimate the double reduction rate when at least one parent is simplex and neither parent is duplex. However, these estimates are biased and have high variance (Figure S13). Our results thus indicate that, though it is important to account for double reduction and preferential pairing when testing for segregation distortion, the estimates of these parameters using just a single biallelic locus are highly unreliable and should not be used in real practical work.

### Alternative simulations

To evaluate our methods, we ran simulations when the alternative was true. We set the true genotype frequencies to be one of the following 14 quantities

13 $$\begin{aligned} \begin{aligned}&\varvec{q} \in \{(1/5,1/5,1/5,1/5,1/5), (1/4, 1/4, 1/4, 1/4, 0),\\&\quad (4/10, 3/10, 2/10, 1/10, 0),\\&\quad (1/10, 2/10, 3/10, 4/10, 0), (1/3, 1/3, 1/3, 0, 0),\\&\quad (0, 1/3, 1/3, 1/3, 0),\\&\quad (3/6, 2/6, 1/6, 0, 0), (0, 3/6, 2/6, 1/6, 0),\\&\quad (1/6, 2/6, 3/6, 0, 0),\\&\quad (0, 1/6, 2/6, 3/6, 0), (3/4, 1/4, 0, 0, 0),\\&\quad (1/4, 3/4, 0, 0, 0),\\&\quad (0, 3/4, 1/4, 0, 0), (0, 1/4, 3/4, 0, 0)\}, \end{aligned} \end{aligned}$$

or sampled uniformly from the 4-simplex. We tested for segregation distortion after estimating $\ell _1$ and $\ell _2$ by maximum likelihood. We varied the sample size $n \in \{20, 200\}$ and the read-depth $\{10, \infty \}$, where a read-depth of $\infty$ corresponds to the known genotype case. Each replication, we simulated offspring genotypes assuming the appropriate $\varvec{q}$ from a multinomial distribution. Our procedure for using genotype likelihoods, and the methods we fit each replication, were the same as in Section Null simulations. For each unique combination of parameter values, we ran 200 replications.

We provide plots of stated type I error versus power for the three methods in Figures S14–S17. Typically (though not always), the chi-squared test is more powerful than polymapR, which is more powerful than the likelihood ratio test. However, only the likelihood ratio test actually controls for type I error, and so we see from this plot that one of the costs of accurately controlling type I error is a loss of power. Though, interestingly, the likelihood ratio test has higher power than polymapR (and even the chi-squared test) in some scenarios.

Box plots for the log Bayes factors are presented in Figure S18. The Bayes factors are generally negative, especially for larger sample sizes, indicating support for the alternative. Though, the Bayes test does not indicate strong support for the alternative when there are three genotypes that each have a true genotype frequency of 1/3. These are also low-power scenarios for the likelihood ratio test (Figures S14–S17). Our Bayes test is relatively robust to prior specification (Appendix S4).

**Fig. 2:**
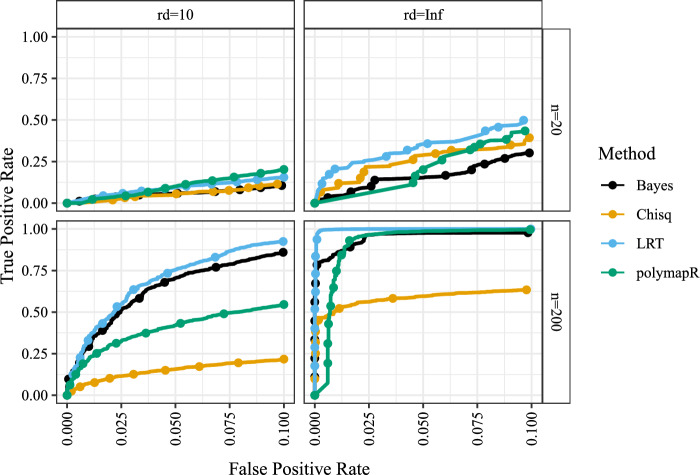
ROC curve at realistic levels of type I error rate. The type I error rate (false positive rate) on the *X*-axis is plotted against power (true positive rate) on the *Y*-axis for various methods (color) across all simulation scenarios: 9000 null and 3000 alternative cases per ROC curve. This is a general overview for the simulation performance of the various methods. The likelihood ratio test is generally the best performer, and the Bayes test is second best for large sample sizes

Since the chi-squared and polymapR tests do not control type I error, unlike our LRT, the power curves in Figures S14–S17 are not directly comparable. We have also yet to perform a direct comparison of the frequentist tests with the Bayesian test. However, it is theoretically possible to calibrate *p*-values or Bayes factors to control type I error by adjusting the rejection thresholds. The performance of the methods would depend on the composition of the null and alternative scenarios, but we can gain an intuitive summary of the performance of the various methods using the null and alternative scenarios that we have studied. We thus combined all 9000 of the null (Section Null simulations) and 3000 of the alternative (Section Alternative simulations) simulation scenarios that we explored in this paper and generated ROC curves (only at realistic levels of type I error) in Fig. 2. We see there at that the likelihood ratio test is the best (or near the best) performing method at all read-depths and sample sizes. The Bayes test is the second best at larger sample sizes. Additionally, Figure S24 confirms that these results remain robust when the alternative scenarios are subsampled to realistic levels of segregation distortion (4%, based on the blueberry data in Section Blueberries). Specifically, for each ROC curve in Figure S24, we randomly sampled 375 of the 3000 alternative scenarios while retaining all 9000 null scenarios. The ROC curves are highly stable across random seeds (results not shown).

### Blueberries

We applied our methods on a dataset of F1 tetraploid blueberries (*Vaccinium corymbosum*) (2n = 4x = 48) from Cappai et al. (2020). The data we considered initially consisted of 21513 SNPs for the $n=240$ offspring and the two parents. We obtained genotype likelihoods using the method of Gerard et al. (2018) with the proportional normal prior (Gerard and Ferrão 2019). Markers were then filtered to remove monomorphic SNPs, defined as those whose maximum genotype frequency was estimated to be greater than 0.95 (20251 remaining SNPs). We then filtered SNPs to keep only loci belonging to the 12 main linkage groups (19524 remaining SNPs). We then ran our LRT (Section Likelihood ratio tests for segregation distortion), our Bayesian test (Section Bayesian tests for segregation distortion), the standard chi-squared test, and the polymapR test for each SNP.

The Bayesian, LRT, and polymapR tests generally agree on the amount of segregation distortion in the data. At a Bonferroni adjusted significance level of 0.05, the LRT and polymapR indicated a segregation distortion rate of 4.4% and 2.6%, respectively. The Bayesian test had 1.6% of SNPs with a log Bayes factor less than -16 (see Wakefield 2010; Gerard 2023, for threshold recommendations for Bayes factors). In contrast, the chi-squared test using posterior mode genotypes indicated 72.8% of SNPs are in segregation distortion, using a Bonferroni corrected significance level of 0.05.

The likelihood ratio and Bayes tests have more concordance on which SNPs indicate segregation distortion (Figure S19). It is enlightening to see which SNPs polymapR and our new methods disagree about. In Figure S20, we provide genotype plots (Gerard et al. 2018) of five SNPs where polymapR indicates no segregation distortion while the LRT indicates extreme segregation distortion. In Figure S21, we provide genotype plots of five SNPs where polymapR indicates extreme segregation distortion while the LRT indicates no segregation distortion. The *p*-values of the various tests for these SNPs are provided in Table S1.

Generally, since polymapR does not account for double reduction, it detects segregation distortion in SNPs that seem to have high rates of double reduction. Examples of these are presented in the last five rows of Table S1. These are all simplex $\times$ nullplex markers that roughly exhibit the 13:10:1 segregation ratios, one would expect at a double reduction rate of $\alpha = 1/6$, and so our LRT and Bayes test correctly indicate that there is no evidence of segregation distortion here. However, at simplex $\times$ nullplex markers, polymapR (and the chi-squared test) assumes a 1:1 segregation ratio and so cannot accommodate offspring genotypes of 2 and segregation ratios beyond 1:1. This leads them to detect segregation distortion at these SNPs.

Conversely, polymapR is more lenient toward “invalid” genotypes, as it only runs its tests on the “valid” genotypes. This leads it to fail to detect segregation distortion at some SNPs where our LRT and Bayes test indicate that there is strong segregation distortion. A few examples of such SNPs are in the first five row in Table S1. At each of these, the tabulated posterior mode genotypes indicate that there are individuals with “invalid” genotypes. E.g., at SNP 12_8929238, genotypes of 3 should be impossible at this simplex $\times$ nullplex marker, even with double reduction, and so our LRT and Bayes test indicate that there is segregation distortion here. However, polymapR’s “valid” genotypes (0 and 1) are at an observed ratio of 106:114, which is close enough to the expected 1:1 ratio that it provides a large *p*-value. The number of “invalid” genotypes is small enough to not be flagged by polymapR. Though, we would argue that observing about 11 “invalid” genotypes (for SNP 12_8929238) should flag possible segregation distortion.

As mentioned in Section Null simulations, the estimates for the double reduction rate are biased and have high variance, and so should not be trusted. However, we can get a sense if our method is performing reasonably by plotting average double reduction rate estimates against the different locations along the linkage groups and seeing if the double reduction rate is generally larger near the ends of the chromosomes (Voorrips and Maliepaard 2012). We averaged the estimated double reduction rate of the first 10%, the middle 20%, and the last 10% of SNPs and plotted these averages (along with plus or minus two standard errors) in Figures S22 and S23. Figure S22 contains SNPs that are simplex for parent 1 and nullplex for parent 2, while Figure S23 contains SNPs that are nullplex for parent 1 and simplex for parent 2. This is so that we can gauge the double reduction estimates for the parents separately without any possible interference from preferential pairing or the other parent. We calculated Tukey adjusted *p*-values (Tukey 1949) comparing the first 10% of SNPs against the middle 20%, and the middle 20% against the last 10%. These *p*-values are posted above the error bars in Figures S22 and S23. We see that many linkage groups, particularly in parent 1, show the middle 20% of SNPs having a lower average double reduction rate than the ends of the linkage groups (linkage groups 1, 2, 4, 20, and 22 in parent 1). In contrast, the only scenario where we have evidence of an end of a chromosome having lower double reduction rates than the middle is linkage group 22 in parent 2. Otherwise, we do not have strong evidence of different values of double reduction between the ends and the middle of the linkage groups. These results at least suggest that our method is picking up some signal of double reduction varying along the chromosome in a way consistent with biological theory.

## Discussion

We developed new models for the gamete frequencies of tetraploids that incorporate both preferential pairing and double reduction. We used these models to develop likelihood ratio and Bayesian tests for segregation distortion in F1 populations that optionally account for genotype uncertainty. We demonstrated that our LRT controls type I error, where competing methods sometimes do not. Our Bayesian test had good performance in simulations, generally supporting the null when the null was true and supporting the alternative when the alternative was true. We demonstrated our methods on a real F1 population of tetraploid blueberries.

Tests for segregation distortion are generally only one part of the quality control pipeline of a study. Indeed, the polymapR package’s checkF1() function performs various checks, of which segregation distortion is one aspect, and aggregates these results into various quality scores. We imagine that our tests derived here could be similarly used as part of a quality control pipeline, where they can be a drop-in replacement for the standard chi-squared test.

Our paper has focused on testing for segregation distortion and not on estimating the meiotic parameters of our new model, the rate of the double reduction and the rate of preferential pairing. We make no claims that our maximum likelihood or Bayes estimates are any good. Indeed, our simulations indicate that the estimates of the double reduction rate have very high variance and bias even for a sample of size 200 (Figure S13), making them useless for practical application. This indicates that there is some theoretical limit in the information at a single biallelic locus to accurately estimate these parameters. Indeed, because of the identifiability issues described in Section Generalized gamete frequencies, it is theoretically impossible to jointly estimate these parameters when a parent is duplex. However, we have shown in this paper that it is important to account for these parameters in the hypothesis test of segregation distortion, even if they cannot be estimated accurately.

Could we adapt our method to use multiple loci to estimate the rate of double reduction and the rate of preferential pairing? It is possible, but we do not think this would be the right approach to estimation. We will detail one possible scheme and then list its shortcomings. First, to not deal with the unidentifiability issues at parental duplex markers (Section Generalized gamete frequencies), we could separately estimate the double reduction rate at loci where at least one parent is simplex and neither parent is duplex. We could not just average these double reduction rate estimates since the double reduction rate is known to vary across the genome (Voorrips and Maliepaard 2012). However, if given a linkage map, we could then use some smoother to improve those estimates. Secondly, we could possibly identify the preferential pairing parameter at loci where the parents are duplex by fixing the double reduction rate to its smoothed estimate at that locus. This would produce estimates of the preferential pairing parameter at duplex loci. Since the preferential pairing parameter is likely fixed within a linkage group (though, see Bourke et al. 2017), we could possibly aggregate all preferential pairing parameter estimates within a linkage group to come up with an estimated preferential pairing rate. Unfortunately, this aggregation would not be simple, as the preferential pairing parameter $\xi$ is defined in terms of $\gamma$, and $\gamma$ is defined as the probability that the two chromosomes that share the same alleles will pair, but which chromosomes share the same alleles likely varies across the genome. Chromosomes 1 and 2 (and, therefore 3 and 4) might share the same allele at one locus, but at other loci chromosomes 1 and 3 (and, therefore 2 and 4) share the same allele, and at yet other loci chromosomes 1 and 4 (and, therefore 2 and 3) share the same allele. The estimated preferential pairing parameters would thus come from three different clusters, and we would have to develop a clustering approach to identify the preferential pairing rate (e.g., see Sun 2020).

Why do we think that such an approach would not work? It does not efficiently account for linkage. There is a lot of information (because of linkage) about the correlation between loci, but the above approach would only use this information in an ad-hoc way via some smoother. A much better approach would be to utilize that linkage information directly, e.g., by some hidden Markov model, as implemented in polymapR (Bourke et al. 2018) or MAPpolly (Mollinari et al. 2020). These softwares have quality control procedures to weed out poorly behaved SNPs before producing their linkage maps, and this is where we see our tests for segregation distortion excelling. These linkage mapping softwares rely on high-quality SNPs, and our new tests for segregation distortion can be used in this context to flag poorly behaved SNPs.

Though the model in Table 2 contains only two parameters, it is not always preferred to that of Table 1 because the ranges of the parameters in the two-parameter model are dependent. This results from the well-known fact that, under various models, there is an upper bound on the rate of double reduction (Mather 1935; Huang et al. 2019). E.g., under the complete equational segregation model, the maximum value of the double reduction rate is 1/6 (so $0 \le \alpha \le \beta \le 1/6$). Suppose that the maximum rate is *c*, then we have by Theorem S2 that

14 $$\begin{aligned} \frac{1}{3}\frac{\alpha }{1-\alpha }\frac{1-c}{c} \le \xi \le 1 - \frac{2}{3}\frac{\alpha }{1-\alpha }\frac{1-c}{c}. \end{aligned}$$

The preferential pairing parameter, $\gamma$, is interpreted as the frequency of bivalent pairing between chromosomes carrying certain alleles. Since individuals might have different alleles on different subgenomes, this has a few consequences for the broader applicability of our model. First, each parent may contain different alleles on different subgenomes, and so each parent should have their preferential pairing parameter modeled separately (either $\gamma$ or $\xi$). Second, as offspring may have different alleles on different subgenomes, this model will not be persistent across more than one F1 population. Thus, it should not naively be used for simulating multiple generations.

Instead of taking a likelihood ratio approach in Section Likelihood ratio tests for segregation distortion, we could have used a chi-squared test statistic for the offspring genotypes against the estimated offspring genotype frequencies under our new model of meiosis. Let $q_k(\hat{\alpha },\hat{\xi }_1,\hat{\xi }_2,\ell _1,\ell _2)$ represent the estimated frequency of offspring genotype $k \in {0,1,2,3,4}$ when there is no segregation distortion. The estimates $\hat{\alpha }$, $\hat{\xi }_1$, and $\hat{\xi }_2$ can be the maximum likelihood estimates as in Section Likelihood ratio tests for segregation distortion (Fisher 1928) or the minimum chi-squared estimates (Neyman 1949; Berkson 1980). The chi-squared test statistic is:

15 $$\begin{aligned} \sum _{k=0}^4\frac{[x_k - n q_k(\hat{\alpha },\hat{\xi }_1,\hat{\xi }_2,\ell _1,\ell _2)]^2}{n q_k(\hat{\alpha },\hat{\xi }_1,\hat{\xi }_2,\ell _1,\ell _2)}. \end{aligned}$$

To obtain the null distribution of this test statistic, we would again have to resort to the adaptive degrees of freedom approach of Susko (2013) since the parameters might lie on (or near) the boundary of the parameter space. Since the likelihood ratio and chi-squared tests are asymptotically equivalent (Lehmann and Romano 2006), and since a likelihood approach can be easily adapted to account for genotype uncertainty while a chi-squared approach cannot be so easily adapted, we chose not to pursue this chi-squared approach.

In some applied scenarios, researchers might know the value of the double reduction rate or the value of the preferential pairing parameter. For example, if researchers know that all pairing is bivalent, then the double reduction rate could be fixed to 0. Additionally, if researchers know that an organism is a true allopolyploid, then they could run two tests (one with $\xi = 0$ and one with $\xi = 1$) and choose the larger of the two *p*-values as the evidence of segregation distortion. Our software implements all of our likelihood ratio and Bayes tests in the cases when (i) only the double reduction rate ($\alpha$) is known, (ii) only the preferential pairing parameters ($\xi _1$ and $\xi _2$) are known, and (iii) both the double reduction rate and the preferential pairing parameters are known.

The methods in this paper are entirely for tetraploids, so a reasonable question would be how feasible an extension to higher ploidies would be? If we only limited ourselves to accounting for double reduction, and not preferential pairing, then we could use the segregation model of Fisher and Mather (1943) and Huang et al. (2019) and develop likelihood ratio and Bayes tests for segregation distortion for arbitrary (even) ploidy levels. If we only limited ourselves to accounting for preferential pairing, and not double reduction (and so only allow for bivalent pairing), then we could use the “configuration” model of Gerard et al. (2018) and develop likelihood ratio and Bayes tests for segregation distortion for arbitrary (even) ploidy levels. Difficulty arises when we want to jointly account for double reduction and preferential pairing. Our tetraploid model is the first to do so at biallelic loci. Extending this to hexaploids and above is non-trivial, and the subject of future work.

## Supplementary Information

Below is the link to the electronic supplementary material.

**Supplementary material** Additional figures, tables, and theoretical details are available in the Supplementary Material online. (pdf 9,192KB)

## Data Availability

The methods described in this paper are implemented in the menbayes package on GitHub: https://github.com/dcgerard/menbayes (https://www.doi.org/10.5281/zenodo.12189055) All analysis scripts and data needed to reproduce the results of this paper are available on GitHub: https://github.com/dcgerard/mbanalysis (https://www.doi.org/10.5281/zenodo.12532001)
