# Supplementary material for: Tests for segregation distortion in tetraploid F1 populations
Source: Theor Appl Genet. 2025 Jan 16;138(1):30. doi: 10.1007/s00122-025-04816-z (PMC11735573; doi:10.1007/s00122-025-04816-z)
Supplement: Supplementary file 1 — Supplementary material Additional figures, tables, and theoretical details are available in the Supplementary Material online. (pdf 9,192KB) [file 122_2025_4816_MOESM1_ESM.pdf]

Supplement to: "Tests for Segregation Distortion in Tetraploid F1 Populations"

David Gerard[1], Mira Thakkar[1], and Luis Felipe V. Ferrão[2]

[1]Department of Mathematics and Statistics, American University, Washington, DC, 20016, USA
[2]Horticultural Sciences Department, University of Florida, Gainesville, FL, 32611, USA

Abstract

This document contains additional theoretical considerations, derivations, and figures to supplement the manuscript "Tests for Segregation Distortion in Tetraploid F1 Populations".

S1 Related work

In this section, we outline prior research on double reduction or preferential pairing in F1 populations. All of these approaches have limitations: (i) none account for both double reduction and preferential pairing at a single biallelic locus simultaneously, (ii) none consider genotype uncertainty, a common issue in polyploid genetics [Gerard et al., 2018, Gerard and Ferrão, 2019], and (iii) most focus on estimating meiotic parameters, rather than testing for segregation distortion, which is our objective.

Many previous approaches have estimated the double reduction rate using gamete frequencies, although the ones mentioned in this paragraph do not consider partial preferential pairing. Fisher and Mather [1943] provides a model for gamete inheritance in the presence of double reduction, but not preferential pairing. This was later generalized to higher ploidies by Huang et al. [2019]. However, neither of these papers provide estimation and testing strategies related to these frequencies. Tai [1982a] and Tai [1982b] estimate the double reduction rate using a complex series of crosses between diploids and tetraploids, but they do not account for preferential pairing, and their scheme was in the context of estimating the quantitative effects of genotypes on phenotypic traits. Haynes and Douches [1993] estimates double reduction, but assumes that one can obtain the gamete genotypes, and also does not account for preferential pairing. Bourke et al. [2015] looked for evidence of double reduction by finding duplex offspring markers in nullplex by simplex crosses but did not provide general methods for more general parental genotypes. Gerard [2022b] estimates double reduction for autopolyploid populations in Hardy-Weinberg equilibrium, but not for F1 populations [see also Gerard, 2022a].

Previous approaches to estimating preferential pairing are not designed for biallelic SNP data or are otherwise limiting. Qu and Hancock [2001] and Bourke et al. [2017] developed methods to estimate the degree of preferential pairing but require simplex by nullplex markers 0 cM apart that are in repulsion linkage. Bourke et al. [2017] can estimate the degree of preferential pairing only when the marker alleles are located on the homologues, but *not* the homoeologues. Cao et al. [2004] also requires simplex by nullplex markers in repulsion linkage but allows for markers to be further than 0 cM apart. The method of Wu et al. [2001] is not designed for biallelic SNP data, assumes equal

preferential pairing at both ends of the chromosome, and provides no software implementing the methods. The model of Wu et al. [2001] further assumes that quadrivalent frequencies of less than 2/3 are the result of preferential pairing, an assumption violated in some organisms such as *Solanum tuberosum* [Swaminathan and Howard, 1953] and *Lotus corniculatus* [Fjellstrom et al., 2001]. Olson [1997] only models random chromosome segregation and disomic segregation, not accounting for double reduction and partial preferential pairing. Sun [2020] provides a model and estimation procedure for preferential pairing for any ploidy but does not account for double reduction. All of these methods also assume that genotypes are known without error, an unrealistic assumption in polyploids [Gerard et al., 2018, Gerard and Ferrão, 2019].

Polyploid phasing software often accounts for preferential pairing or double reduction, and so the degree of preferential pairing or the double reduction rate are estimated as a by-product [Zheng et al., 2016, Bourke et al., 2018, Mollinari et al., 2020, Zheng et al., 2021]. However, such methods either do not allow for heterogeneous levels of preferential pairing [Zheng et al., 2016, Bourke et al., 2018, Zheng et al., 2021], or do not accommodate double reduction [Bourke et al., 2018, Mollinari et al., 2020], which we show in Section 4 can be confounded with the effects of preferential pairing.

One paper that does account for both double reduction and preferential pairing in tetraploids is Stift et al. [2008]. This paper provides a general model for chromosome segregation in tetraploids with arbitrary levels of double reduction and preferential pairing. However, the model of Stift et al. [2008] assumes that all chromosomes are uniquely marked, and that gametes can be uniquely genotyped. This is not typically the case for modern biallelic SNP markers. In Section S2, we use the model of Stift et al. [2008] as a starting point to derive a model for gamete frequencies that are agnostic to chromosomal assignment, and may thus be applied to biallelic SNP data.

S2 A model for gamete frequencies, accounting for double reduction and preferential pairing

Stift et al. [2008] proposed a model for segregation in tetraploids that accounts for both preferential pairing and double reduction. This model assumes that all four chromosomes can be distinguished. However, researchers typically only have biallelic dosage data (i.e. the number of copies of the alternative allele for an individual at a locus) [Gerard et al., 2018, Gerard and Ferrão, 2019] where each chromosome cannot be separately determined. Here, we will modify the model of Stift et al. [2008] over chromosome assignment to derive a model for segregation at biallelic loci in tetraploids.

We begin by describing the model of Stift et al. [2008]. Let c_1, c_2, c_3, and c_4 denote the four chromosomes of a tetraploid individual. The model of Stift et al. [2008] has three parameters. Let τ be the proportion of quadrivalent pairing, let β be the probability of double reduction given quadrivalent pairing, so $\alpha = \tau\beta$ is the double reduction rate, and let $(\delta_1, \delta_2, \delta_3)$ be the rates of different bivalent pairings, where δ_1 is the probability of c_1 and c_2 pairing together given bivalent formation, δ_2 is the probability of c_1 and c_3 pairing together given bivalent formation, and δ_3 is the probability of c_1 and c_4 pairing together given bivalent formation.. Note that $\delta_1 + \delta_2 + \delta_3 = 1$ and, for identifiability reasons, Stift et al. [2008] constrain $\delta_1\delta_2\delta_3 = 0$ (at least one pairing has zero probability), but we don't make this identifying assumption. Then the model of Stift et al. [2008]

states the probability of each gamete to be

$$\begin{pmatrix} \Pr(c_1c_1) \\ \Pr(c_2c_2) \\ \Pr(c_3c_3) \\ \Pr(c_4c_4) \\ \Pr(c_1c_2) \\ \Pr(c_1c_3) \\ \Pr(c_1c_4) \\ \Pr(c_3c_4) \\ \Pr(c_2c_4) \\ \Pr(c_2c_3) \end{pmatrix} = \begin{pmatrix} 0 \\ 0 \\ 0 \\ 0 \\ 1/6 \\ 1/6 \\ 1/6 \\ 1/6 \\ 1/6 \\ 1/6 \end{pmatrix} \tau + \begin{pmatrix} 1/4 \\ 1/4 \\ 1/4 \\ 1/4 \\ -1/6 \\ -1/6 \\ -1/6 \\ -1/6 \\ -1/6 \\ -1/6 \end{pmatrix} \beta\tau + (1-\tau) \begin{pmatrix} 0 & 0 & 0 \\ 0 & 0 & 0 \\ 0 & 0 & 0 \\ 0 & 0 & 0 \\ 0 & 1/4 & 1/4 \\ 1/4 & 0 & 1/4 \\ 1/4 & 1/4 & 0 \\ 0 & 1/4 & 1/4 \\ 1/4 & 0 & 1/4 \\ 1/4 & 1/4 & 0 \end{pmatrix} \begin{pmatrix} \delta_1 \\ \delta_2 \\ \delta_3 \end{pmatrix}. \tag{S1}$$

More concisely, equation (S1) may be written as

$$Pr(c_1c_1) = Pr(c_2c_2) = Pr(c_3c_3) = Pr(c_4c_4) = \frac{1}{4}\beta\tau, \tag{S2}$$

$$Pr(c_1c_2) = Pr(c_3c_4) = \frac{1}{6}\tau(1-\beta) + \frac{1}{4}(1-\tau)(\delta_2+\delta_3), \tag{S3}$$

$$Pr(c_1c_3) = Pr(c_2c_4) = \frac{1}{6}\tau(1-\beta) + \frac{1}{4}(1-\tau)(\delta_1+\delta_3), \tag{S4}$$

$$Pr(c_1c_4) = Pr(c_2c_3) = \frac{1}{6}\tau(1-\beta) + \frac{1}{4}(1-\tau)(\delta_1+\delta_2). \tag{S5}$$

We will now modify model (S2)–(S5) to biallelic dosage data. Let $\ell \in \{0,1,2,3,4\}$ be the dosage of the parent at a locus, and let $x \in \{0,1,2\}$ be the random variable of the number of alternative alleles that the parent sends to an offspring. We assume that we do not know which chromosomes contain the alternative and reference alleles. Then, for a parental dosage of $\ell = 0$, none of the chromosomes have the reference allele, and we have

$$\begin{aligned} Pr(x=0|\ell=0) &= Pr(c_1c_2) + Pr(c_1c_3) + Pr(c_1c_4) + Pr(c_2c_3) + Pr(c_2c_4) \\ &\quad + Pr(c_3c_4) + Pr(c_1c_1) + Pr(c_2c_2) + Pr(c_3c_3) + Pr(c_4c_4) \qquad \text{(S6)} \\ &= 1 \end{aligned}$$

$$Pr(x=1|\ell=0) = Pr(x=2|\ell=0) = 0. \tag{S7}$$

By symmetry, we have

$$Pr(x=0|\ell=4) = Pr(x=1|\ell=4) = 0 \tag{S8}$$

$$Pr(x=2|\ell=4) = 1. \tag{S9}$$

For a parental dosage of $\ell = 1$, for the moment allow c_1 to carry the A allele, and c_2, c_3, and c_4 to carry the a allele. Then

$$Pr(x=0|\ell=1) = Pr(c_2c_3) + Pr(c_2c_4) + Pr(c_3c_4) + Pr(c_2c_2) + Pr(c_3c_3) + Pr(c_4c_4) \tag{S10}$$

$$= \frac{1}{2}\tau(1-\beta) + \frac{1}{4}(1-\tau)(\delta_1+\delta_2+\delta_1+\delta_3+\delta_2+\delta_3) + \frac{3}{4}\beta\tau \tag{S11}$$

$$= \frac{1}{2}\tau(1-\beta) + \frac{1}{2}(1-\tau) + \frac{3}{4}\beta\tau \tag{S12}$$

$$= \frac{1}{2} + \frac{1}{4}\beta\tau. \tag{S13}$$

$$Pr(x = 1|\ell = 1) = Pr(c_1c_2) + Pr(c_1c_3) + Pr(c_1c_4) \tag{S14}$$

$$= \frac{1}{2}\tau(1-\beta) + \frac{1}{4}(1-\tau)(\delta_2 + \delta_3 + \delta_1 + \delta_3 + \delta_1 + \delta_2) \tag{S15}$$

$$= \frac{1}{2}\tau(1-\beta) + \frac{1}{2}(1-\tau) \tag{S16}$$

$$= \frac{1}{2} - \frac{1}{2}\beta\tau \tag{S17}$$

$$Pr(x = 2|\ell = 1) = Pr(c_1c_1) = \frac{1}{4}\beta\tau. \tag{S18}$$

This probability is independent of the labeling for which chromosome carries the A allele, and so is the probability distribution of a gamete dosage given the parental dosage. By symmetry, we have

$$Pr(x = 0|\ell = 3) = \frac{1}{4}\beta\tau, \tag{S19}$$

$$Pr(x = 1|\ell = 3) = \frac{1}{2} - \frac{1}{2}\beta\tau \tag{S20}$$

$$Pr(x = 2|\ell = 3) = \frac{1}{2} + \frac{1}{4}\beta\tau. \tag{S21}$$

For a parental dosage of $\ell = 2$, for the moment allow c_1 and c_2 to carry the A allele, and c_3 and c_4 to carry the a allele. Then

$$Pr(x = 0|\ell = 2) = Pr(c_3c_4) + Pr(c_3c_3) + Pr(c_4c_4) \tag{S22}$$

$$= \frac{1}{6}\tau(1-\beta) + \frac{1}{4}(1-\tau)(\delta_2 + \delta_3) + \frac{1}{2}\beta\tau \tag{S23}$$

$$= \frac{1}{3}\beta\tau + \frac{1}{6}\tau + \frac{1}{4}(1-\tau)(1-\delta_1). \tag{S24}$$

By symmetry we have

$$Pr(x = 2|\ell = 2) = \frac{1}{3}\beta\tau + \frac{1}{6}\tau + \frac{1}{4}(1-\tau)(1-\delta_1). \tag{S25}$$

Finally,

$$Pr(x = 1|\ell = 2) = Pr(c_1c_3) + Pr(c_1c_4) + Pr(c_2c_3) + Pr(c_2c_4) \tag{S26}$$

$$= \frac{2}{3}\tau(1-\beta) + \frac{1}{4}(1-\tau)(\delta_1 + \delta_3 + \delta_1 + \delta_2 + \delta_1 + \delta_2 + \delta_1 + \delta_3) \tag{S27}$$

$$= -\frac{2}{3}\beta\tau + \frac{2}{3}\tau + \frac{1}{2}(1-\tau)(1+\delta_1). \tag{S28}$$

Notice that δ_1 is the probability that the chromosomes that share the same alleles will pair (c_1 with c_2 and c_3 with c_4). To make these probabilities independent of the labeling of the chromosomes, we set γ to be the probability that the chromosomes that share the same alleles will pair, obtaining

$$Pr(x = 0|\ell = 2) = Pr(x = 2|\ell = 2) = \frac{1}{3}\beta\tau + \frac{1}{6}\tau + \frac{1}{4}(1-\tau)(1-\gamma), \text{ and} \tag{S29}$$

$$Pr(x = 1|\ell = 2) = -\frac{2}{3}\beta\tau + \frac{2}{3}\tau + \frac{1}{2}(1-\tau)(1+\gamma), \tag{S30}$$

where $\gamma \in \{\delta_1, \delta_2, \delta_3\}$. We summarize the probabilities of $Pr(x|\ell)$ in Table 1.

The model in 1 contains three parameters. However, we can reduce it down to two parameters. Let η be the probability of quadrivalent formation given no double reduction. That is, using Bayes rule,

$$\eta = \frac{(1-\beta)\tau}{(1-\beta)\tau + (1-\tau)}. \tag{S31}$$

Let ξ be a convex combination between γ and 1/3, weighted by η

$$\xi = \eta\frac{1}{3} + (1-\eta)\gamma. \tag{S32}$$

This ξ parameter measures the degree of preferential pairing, where deviations from 1/3 represent deviations from autopolyploidy, while deviations from 1 or 0 represent deviations from allopolyploidy. Finally, let α be the marginal probability of double reduction

$$\alpha = \beta\tau. \tag{S33}$$

Then, using the parameters α and ξ, we may re-write the probability distributions in Table 1 as Table 2 (Theorem S1).

Theorem S1. *Let ξ and α be as defined in (S32) and (S33), respectively. Then the probability distributions in Tables 1 and 2 are equivalent.*

Proof. The correspondence between $Pr(x|\ell = 0)$, $Pr(x|\ell = 1)$, $Pr(x|\ell = 3)$, and $Pr(x|\ell = 4)$ between the two tables is obvious. It suffices to show that

$$\frac{1}{2}(1-\alpha)(1+\xi) = -\frac{2}{3}\beta\tau + \frac{2}{3}\tau + \frac{1}{2}(1-\tau)(1+\gamma), \tag{S34}$$

as the other equalities from $Pr(x|\ell = 2)$ would follow by the sum-to-one constraint. From the left-hand side of (S34), we have

$$\frac{1}{2}(1-\alpha)(1+\xi) = \frac{1}{2}(1-\beta\tau)(1+\eta\frac{1}{3} + (1-\eta)\gamma) \tag{S35}$$

$$= \frac{1}{2}(1-\beta\tau)\left(1 + \frac{\frac{1}{3}(1-\beta)\tau}{(1-\beta)\tau + (1-\tau)} + \frac{(1-\tau)\gamma}{(1-\beta)\tau + (1-\tau)}\right) \tag{S36}$$

$$= \frac{1}{2}(1-\beta\tau)\left(\frac{(1-\beta)\tau + (1-\tau) + \frac{1}{3}(1-\beta)\tau + (1-\tau)\gamma}{(1-\beta)\tau + (1-\tau)}\right) \tag{S37}$$

$$= \frac{1}{2}(1-\beta\tau)\left(\frac{\frac{4}{3}(1-\beta)\tau + (1-\tau)(1+\gamma)}{(1-\beta)\tau + (1-\tau)}\right) \tag{S38}$$

$$= \frac{1}{2}(1-\beta\tau)\left(\frac{\frac{4}{3}(1-\beta)\tau + (1-\tau)(1+\gamma)}{1-\beta\tau}\right) \tag{S39}$$

$$= \frac{2}{3}(1-\beta)\tau + \frac{1}{2}(1-\tau)(1+\gamma) \tag{S40}$$

$$= -\frac{2}{3}\beta\tau + \frac{2}{3}\tau + \frac{1}{2}(1-\tau)(1+\gamma). \tag{S41}$$

□

Though we have managed to reduce the number of parameters from three to two, the resulting parameterization induces a dependence on the range of possible value of ξ given a value of α (Theorem S2).

Theorem S2. *Suppose that $0 \leq \beta \leq c$. Then, for a given value of α, we have*

$$\frac{1}{3}\frac{\alpha}{1-\alpha}\frac{1-c}{c} \leq \xi \leq 1 - \frac{2}{3}\frac{\alpha}{1-\alpha}\frac{1-c}{c}. \tag{S42}$$

Proof. We may rewrite ξ as

$$\xi = \frac{1}{3}\frac{\tau-\beta\tau}{1-\beta\tau} + \frac{1-\tau}{1-\beta\tau}\gamma \tag{S43}$$

$$= \frac{1}{3}\frac{\tau-\alpha}{1-\alpha} + \frac{1-\tau}{1-\alpha}\gamma. \tag{S44}$$

Since $\tau = \alpha/\beta$ (and $\alpha \leq \beta$), we have, for a given value of α, that $\alpha/c \leq \tau \leq 1$.

To find the minimum value of ξ, we minimize (S44) over τ and γ. This minimum occurs at $\gamma = 0$ and $\tau = \alpha/c$, yielding the lower bound of (S42). To find the maximum value of ξ, we maximize (S44) over τ and γ. This maximum occurs at $\gamma = 1$ and $\tau = \alpha/c$, yielding the upper bound of (S42). □

S3 Generalization of Fisher and Mather [1943]

Here, we show that our models for the gamete frequencies in Table 2 (and, thus, Table 1) are generalizations of those in Table 9 from Fisher and Mather [1943]. The model from Fisher and Mather [1943] incorporates double reduction, but not preferential pairing, and we show that setting $\xi = 1/3$ results in the same gamete frequencies as those in Table 9 from Fisher and Mather [1943]. It is trivial to check the equivalence between Table 2 and Table 9 from Fisher and Mather [1943] for the parental genotypes $\ell = 0, 1, 3, 4$, so we just consider $\ell = 2$. For our tetraploid model, we have that ξ is the probability of pairing configuration A-A:a-a, and $1-\xi$ is the probability of pairing configuration A-a:A-a. Labeling c_1, c_2, c_3, c_4 as the four chromosomes, and allowing c_1 and c_2 to carry the A alleles, the possible pairings are c_1-c_2:c_3-c_4 = A-A;a-a, c_1-c_3:c_2-c_4 = A-a:A-a, and c_1-c_4:c_2-c_3 = A-a:A-a. Thus, under polysomic inheritance, A-A:a-a occurs with probability $\xi = 1/3$. We can plug this in to get the gamete frequencies under polysomic inheritance

$$\Pr(x = 0|\ell = 2, \xi = 1/3) = \frac{1}{2}\alpha + \frac{1}{4}(1 - \frac{1}{3})(1-\alpha) \tag{S45}$$

$$= \frac{1}{6} + \frac{1}{3}\alpha, \tag{S46}$$

which one can check is the same as the value as Table 9 from Fisher and Mather [1943]. The other values for $\ell = 2$ follow from symmetry and the sum-to-one constraint.

S4 Prior Sensitivity Analysis

In this section, we adjusted the default priors for the Bayes test in Section 2.3 to evaluate its robustness to prior selection. Specifically, under the same simulation settings as Sections 3.1 and 3.2, we evaluated our methods using the following priors under the null,

$$\tau \sim \text{Beta}(1/2, 1/2) \text{ or } \tau \sim \text{Beta}(2, 2), \text{ and} \tag{S47}$$

$$\gamma_1, \gamma_2 \sim \text{Beta}(1/3, 2/3) \text{ or } \gamma_1, \gamma_2 \sim \text{Beta}(1, 2). \tag{S48}$$

This resulted in four combinations of new prior distributions (two for τ and two for γ_1 and γ_2). The Beta$(1/3, 2/3)$ prior for the γ's is more informative for allopolyploidy, while the Beta$(1, 2)$ prior for the γ's is more informative for autopolyploidy. The Beta$(1/2, 1/2)$ prior for τ places more weight at either fully bivalent or fully quadrivalent formation, while the Beta$(2, 2)$ prior for τ places more weight on a mixed form of pairing (both bivalent and quadrivalent).

The results for the null simulations of Section 3.1 are presented in Figures S9–S12. We see that the Bayes test is robust to moderate changes in prior selection in almost all scenarios. The only scenario where we see a lot of impact is when $\alpha = 0$, $\xi_1 = \xi_2 = 1$, and $\ell_1 = \ell_2 = 2$. This is the scenario of "fixed heterozygosity" [Cornille et al., 2016] in true allopolyploids where the genotype frequencies are $\boldsymbol{q} = (0, 0, 1, 0, 0)$. That is, all offspring have a genotype of 2. Here, the best performing prior (in terms of having the largest Bayes factors) in this fixed heterozygosity case is the one most keen toward allopolyploidy and pure bivalent pairing ($\gamma_1, \gamma_2 \sim \text{Beta}(1/3, 2/3)$ and $\tau \sim \text{Beta}(1/2, 1/2)$). If fixed heterozygosity were a possibility, this would probably be known by the researcher and they should use a prior that is highly informative for allopolyploidy.

The results for the alternative simulations of Section 3.2 are presented in Figure S18. We see there that all priors performed about equally well, indicating that our methods are robust here to prior selection.

S5 Supplementary tables, figures, and procedures

SNP	ℓ_1	ℓ_2	Bayes	LRT	polymapR	Observed	Expected
12_8929238	0	1	-35	3.9e-22	0.97	(105,113,11,11,0)	(130,100,10,0,0)
11_32341161	2	4	-16	1.8e-15	0.81	(0,8,35,163,34)	(0,0,38.8,162.3,38.8)
6_15037920	0	2	-16	6.2e-14	0.96	(36,153,44,7,0)	(42.7,154.6,42.7,0,0)
6_14723914	4	2	-13	4.8e-12	0.84	(0,7,40,156,37)	(0,0,42.1,155.8,42.1)
17_9734135	0	3	-6.5	2.1e-10	0.83	(23,102,108,6,1)	(10,100,130,0,0)
1_1019768	1	0	11	0.97	0.0016	(132,100,8,0,0)	(130,100,10,0,0)
22_23904806	0	1	11	0.88	0.002	(134,97,9,0,0)	(130,100,10,0,0)
2_12659258	1	0	11	0.99	0.002	(131,101,8,0,0)	(130,100,10,0,0)
2_12659201	1	0	11	0.98	0.002	(131,101,8,0,0)	(130,100,10,0,0)
12_30140888	0	1	9.9	0.89	0.0031	(132,97,11,0,0)	(130,100,10,0,0)

Table S1: In the first five SNPs (plotted in Figure S20), the LRT indicates segregation distortion while the polymapR test indicates no segregation distortion, while in the last five SNPs (plotted in Figure S21) the LRT indicates no segregation distortion while the polymapR test indicates segregation distortion. Parent genotypes (ℓ_1 and ℓ_2) are listed, along with the log Bayes factors ("Bayes"), the LRT p-values ("LRT"), the polyampR p-values ("polymapR"), the observed counts when tabulating posterior mode genotypes ("Observed"), and the expected counts based on the maximum likelihood estimates of α and the ξ's ("Expected").

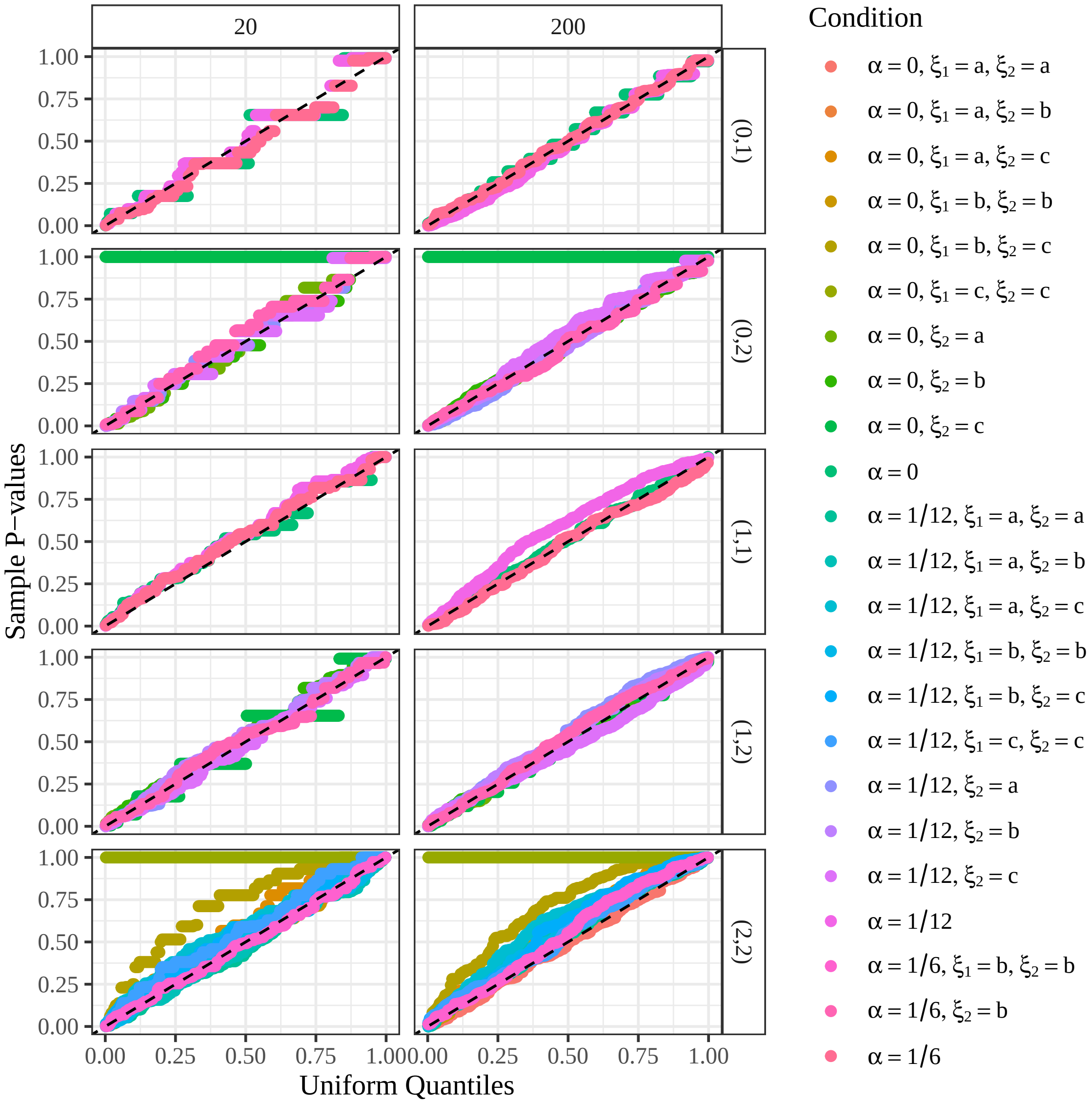

Figure S1: Quantile-quantile plots against the uniform distribution of the p-values of the likelihood ratio test of Section 2.2, when using known genotypes, from the null simulations in Section 3.1. Since the null is true, the points should lie either near or above the $y = x$ line (black dashed line). Points that are above the $y = x$ line are conservative, points that are below the $y = x$ line are anti-conservative. Row-facets index parent genotypes (ℓ_1, ℓ_2), and column-facets index sample size. Color indexes different values of the double reduction rate, α, and the preferential pairing parameters of the two parents, ξ_1 and ξ_2. A preferential pairing value of "a" indicates the lower bound in (S42), a value of "b" indicates 1/3, and a value of "c" indicates the upper bound in (S42). Preferential pairing only affects offspring genotype frequencies when the parent genotype is 2, and so an omission of ξ_1 or ξ_2 from the color legend indicates a scenario where the corresponding parent genotype is not 2.

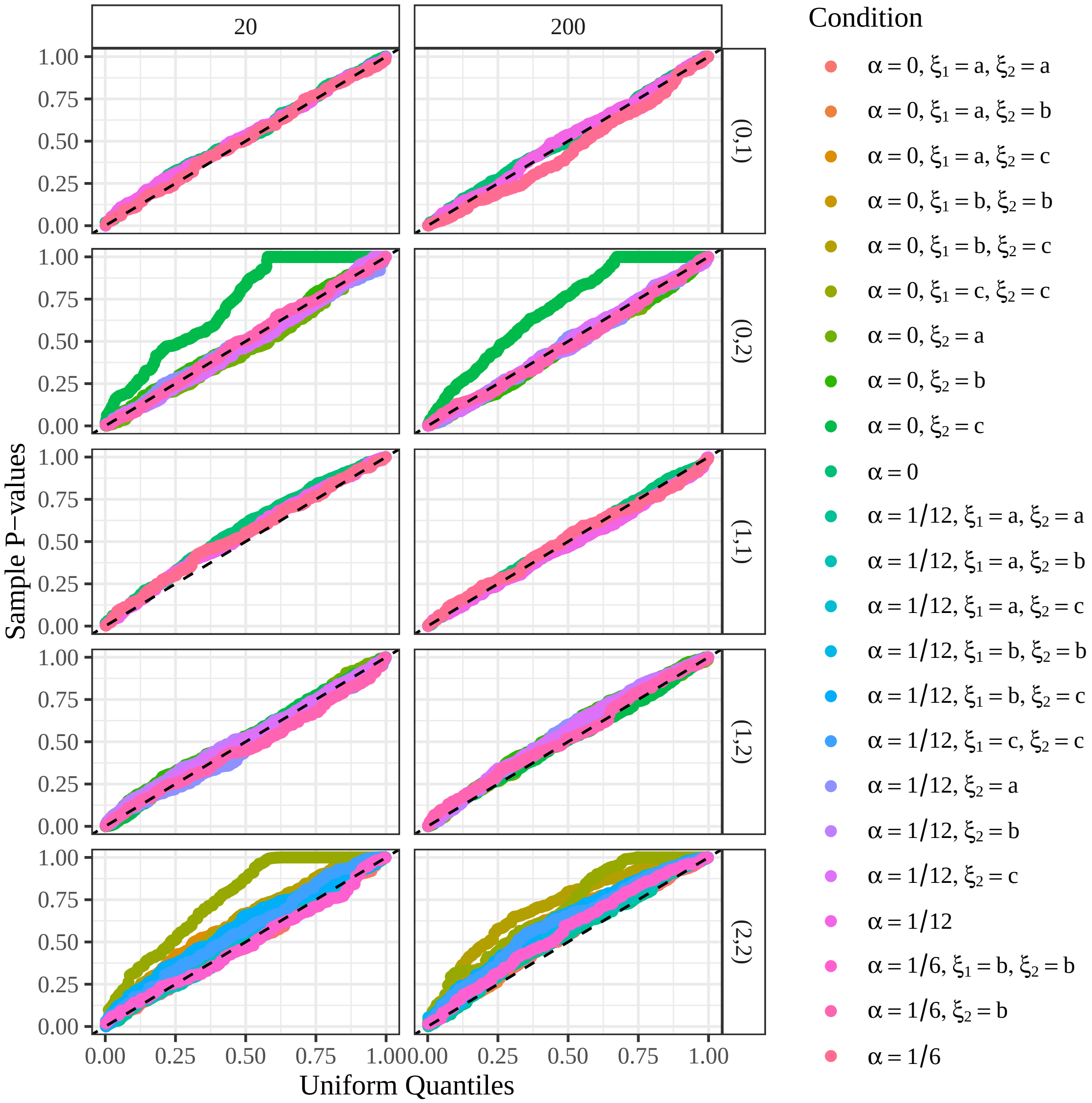

Figure S2: Quantile-quantile plots against the uniform distribution of the p-values of the likelihood ratio test of Section 2.2, using genotype likelihoods derived from read-counts simulated at a read-depth of 10, from the null simulations in Section 3.1. Since the null is true, the points should lie either near or above the $y = x$ line (black dashed line). Points that are above the $y = x$ line are conservative, points that are below the $y = x$ line are anti-conservative. Row-facets index parent genotypes (ℓ_1, ℓ_2), and column-facets index sample size. Color indexes different values of the double reduction rate, α, and the preferential pairing parameters of the two parents, ξ_1 and ξ_2. A preferential pairing value of "a" indicates the lower bound in (S42), a value of "b" indicates 1/3, and a value of "c" indicates the upper bound in (S42). Preferential pairing only affects offspring genotype frequencies when the parent genotype is 2, and so an omission of ξ_1 or ξ_2 from the color legend indicates a scenario where the corresponding parent genotype is not 2.

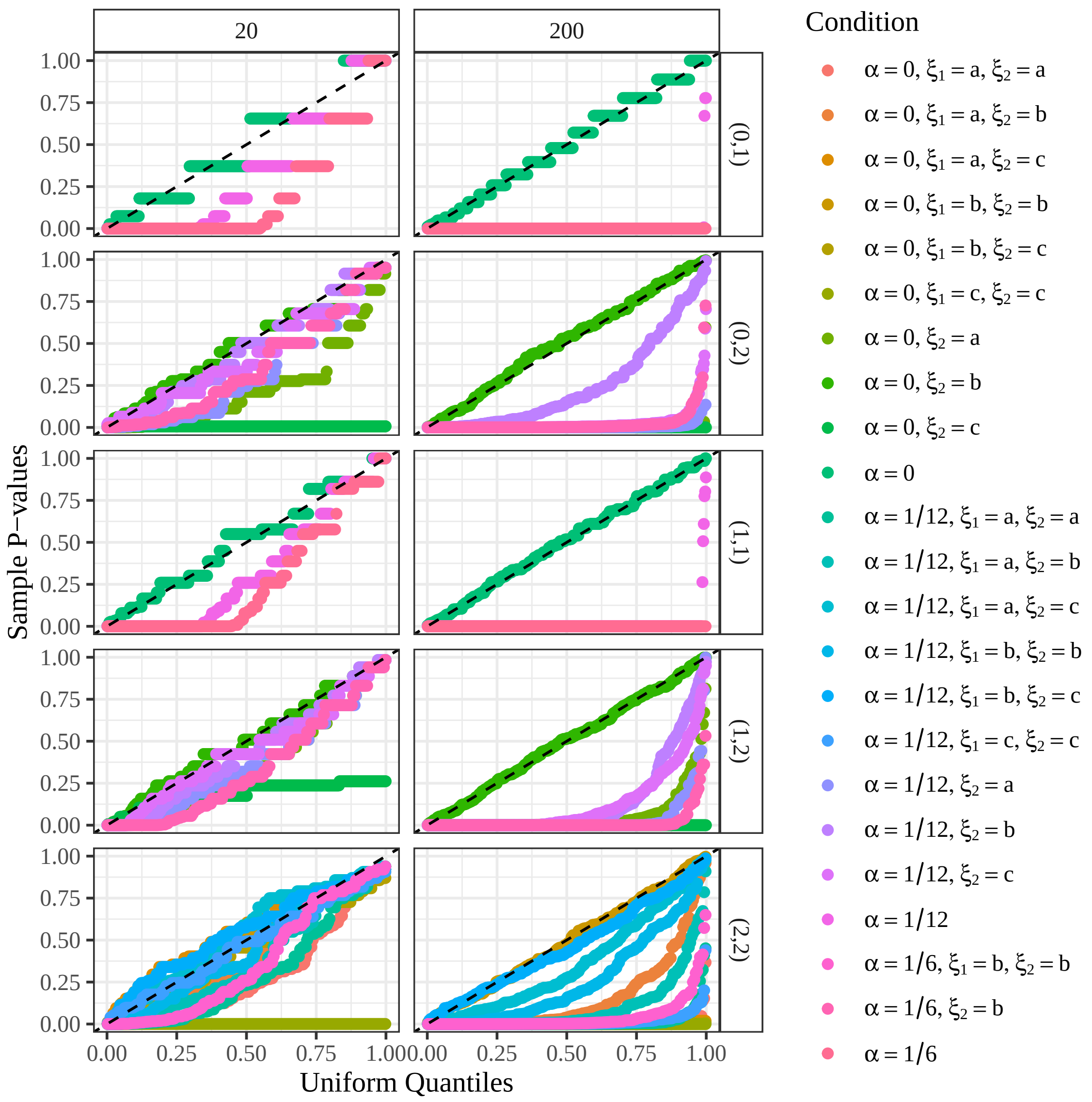

Figure S3: Quantile-quantile plots against the uniform distribution of the p-values of the standard chi-squared test for segregation distortion, when using known genotypes, from the null simulations in Section 3.1. Since the null is true, the points should lie either near or above the $y = x$ line (black dashed line). Points that are above the $y = x$ line are conservative, points that are below the $y = x$ line are anti-conservative. Row-facets index parent genotypes (ℓ_1, ℓ_2), and column-facets index sample size. Color indexes different values of the double reduction rate, α, and the preferential pairing parameters of the two parents, ξ_1 and ξ_2. A preferential pairing value of “a” indicates the lower bound in (S42), a value of “b” indicates 1/3, and a value of “c” indicates the upper bound in (S42). Preferential pairing only affects offspring genotype frequencies when the parent genotype is 2, and so an omission of ξ_1 or ξ_2 from the color legend indicates a scenario where the corresponding parent genotype is not 2.

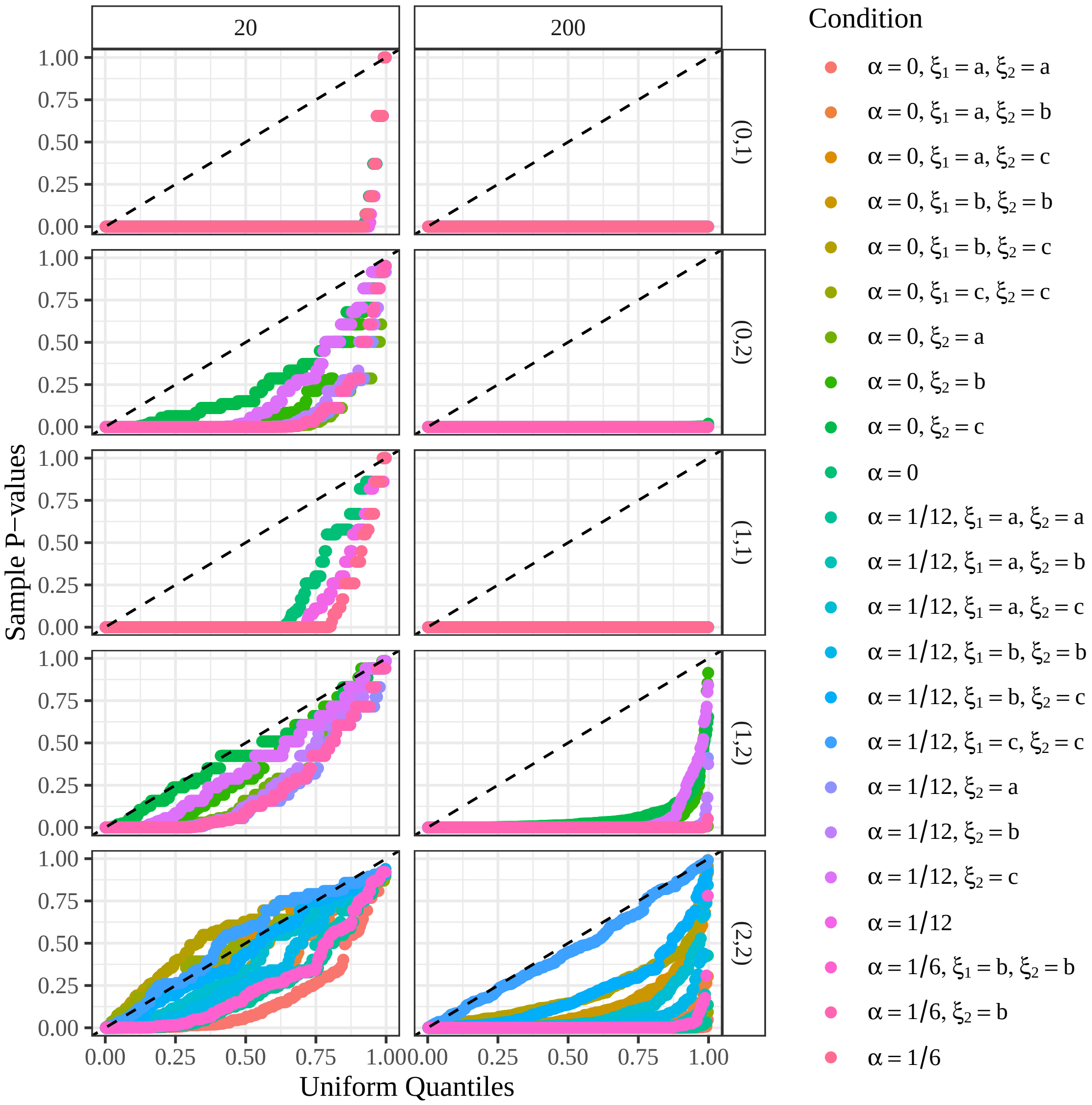

Figure S4: Quantile-quantile plots against the uniform distribution of the p-values of the standard chi-squared test for segregation distortion, using genotype likelihoods derived from read-counts simulated at a read-depth of 10, and tabulating posterior mode genotypes, from the null simulations in Section 3.1. Since the null is true, the points should lie either near or above the $y = x$ line (black dashed line). Points that are above the $y = x$ line are conservative, points that are below the $y = x$ line are anti-conservative. Row-facets index parent genotypes (ℓ_1, ℓ_2), and column-facets index sample size. Color indexes different values of the double reduction rate, α, and the preferential pairing parameters of the two parents, ξ_1 and ξ_2. A preferential pairing value of "a" indicates the lower bound in (S42), a value of "b" indicates 1/3, and a value of "c" indicates the upper bound in (S42). Preferential pairing only affects offspring genotype frequencies when the parent genotype is 2, and so an omission of ξ_1 or ξ_2 from the color legend indicates a scenario where the corresponding parent genotype is not 2.

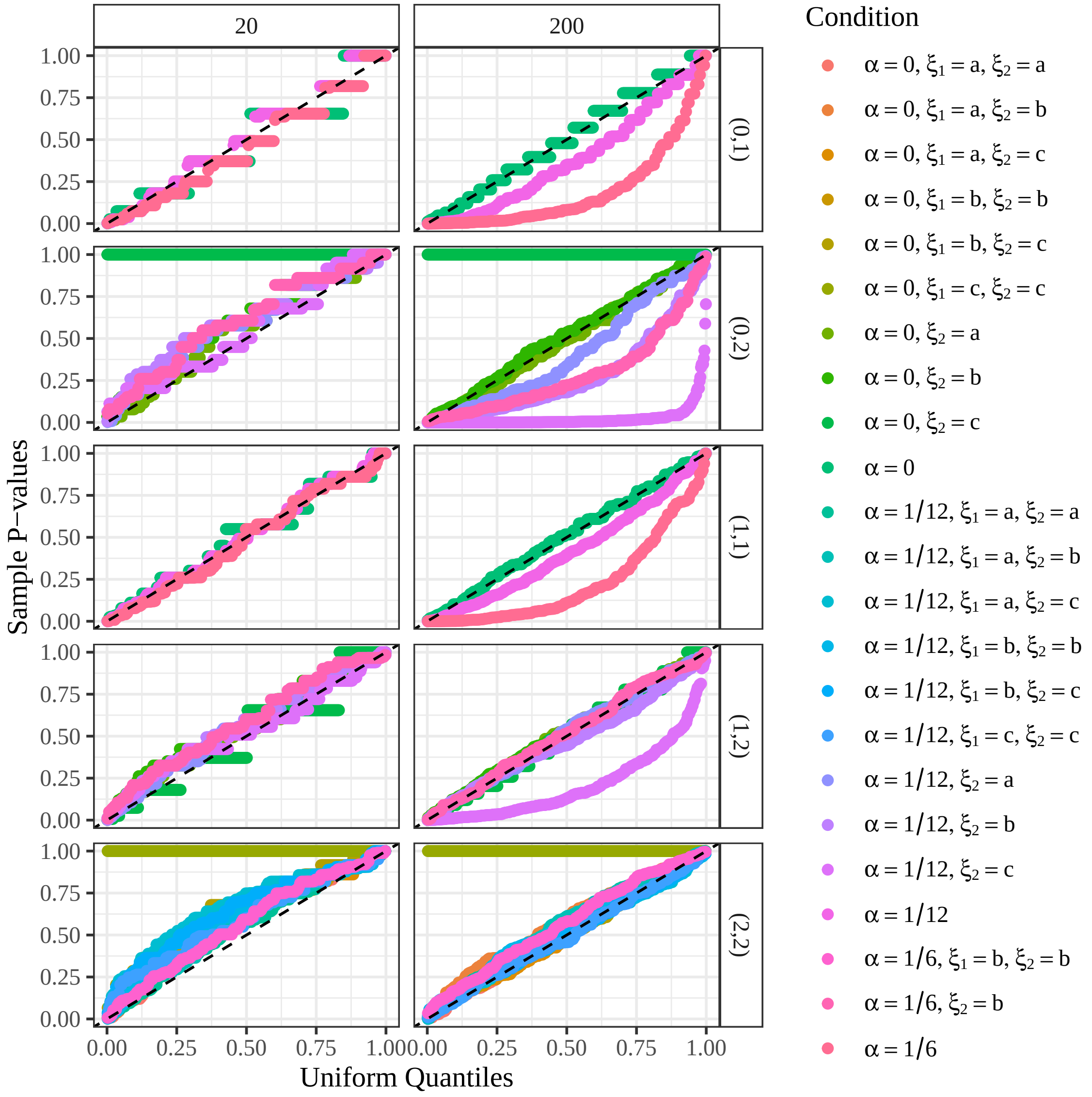

Figure S5: Quantile-quantile plots against the uniform distribution of the p-values of the polymapR approach of Section 1.1 [Bourke et al., 2018], when using known genotypes, from the null simulations in Section 3.1. Since the null is true, the points should lie either near or above the $y = x$ line (black dashed line). Points that are above the $y = x$ line are conservative, points that are below the $y = x$ line are anti-conservative. Row-facets index parent genotypes (ℓ_1, ℓ_2), and column-facets index sample size. Color indexes different values of the double reduction rate, α, and the preferential pairing parameters of the two parents, ξ_1 and ξ_2. A preferential pairing value of “a” indicates the lower bound in (S42), a value of “b” indicates 1/3, and a value of “c” indicates the upper bound in (S42). Preferential pairing only affects offspring genotype frequencies when the parent genotype is 2, and so an omission of ξ_1 or ξ_2 from the color legend indicates a scenario where the corresponding parent genotype is not 2.

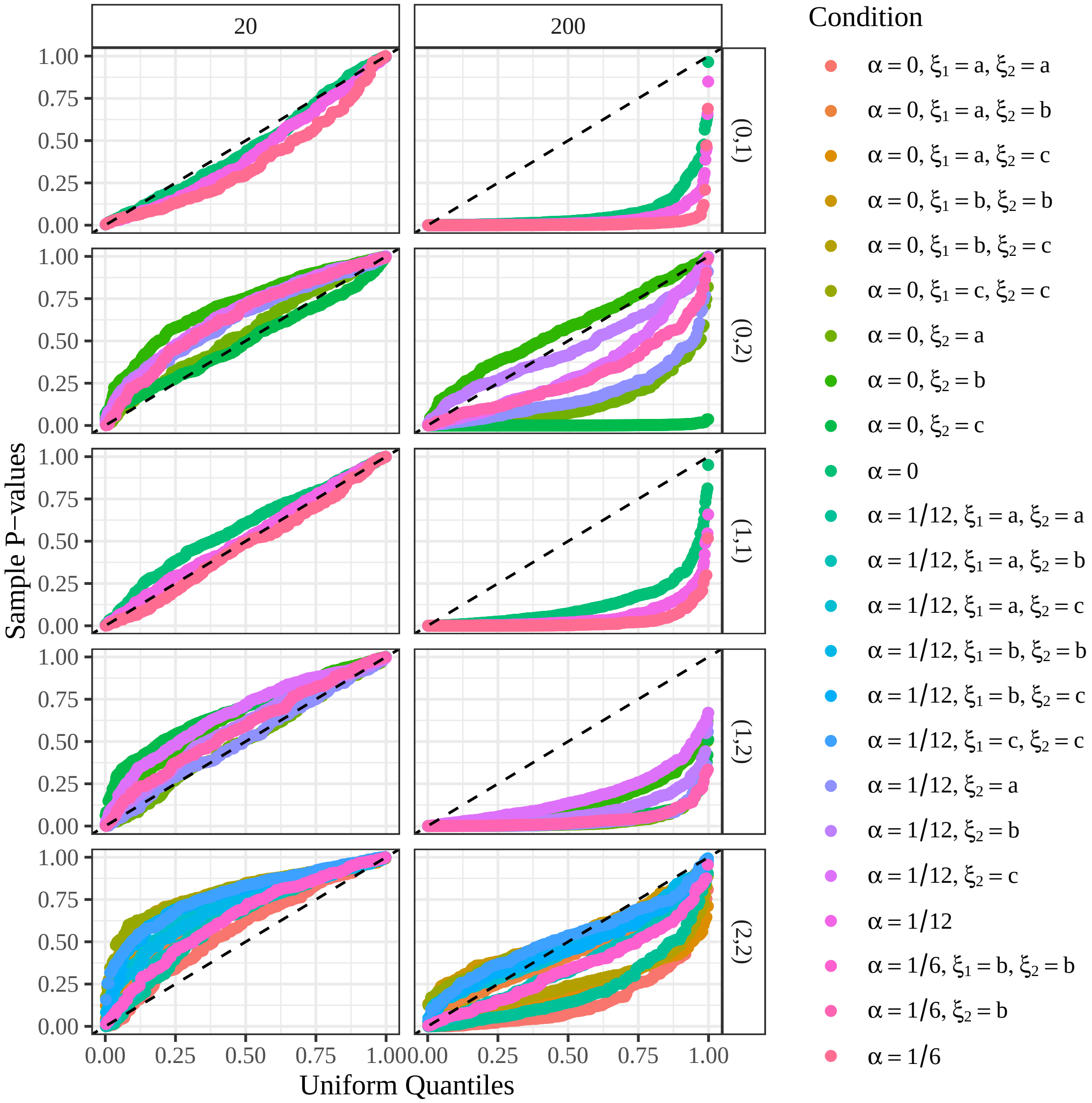

Figure S6: Quantile-quantile plots against the uniform distribution of the p-values of the polymapR approach of Section 1.1 [Bourke et al., 2018], using genotype likelihoods derived from read-counts simulated at a read-depth of 10, from the null simulations in Section 3.1. Since the null is true, the points should lie either near or above the $y = x$ line (black dashed line). Points that are above the $y = x$ line are conservative, points that are below the $y = x$ line are anti-conservative. Row-facets index parent genotypes (ℓ_1, ℓ_2), and column-facets index sample size. Color indexes different values of the double reduction rate, α, and the preferential pairing parameters of the two parents, ξ_1 and ξ_2. A preferential pairing value of "a" indicates the lower bound in (S42), a value of "b" indicates 1/3, and a value of "c" indicates the upper bound in (S42). Preferential pairing only affects offspring genotype frequencies when the parent genotype is 2, and so an omission of ξ_1 or ξ_2 from the color legend indicates a scenario where the corresponding parent genotype is not 2.

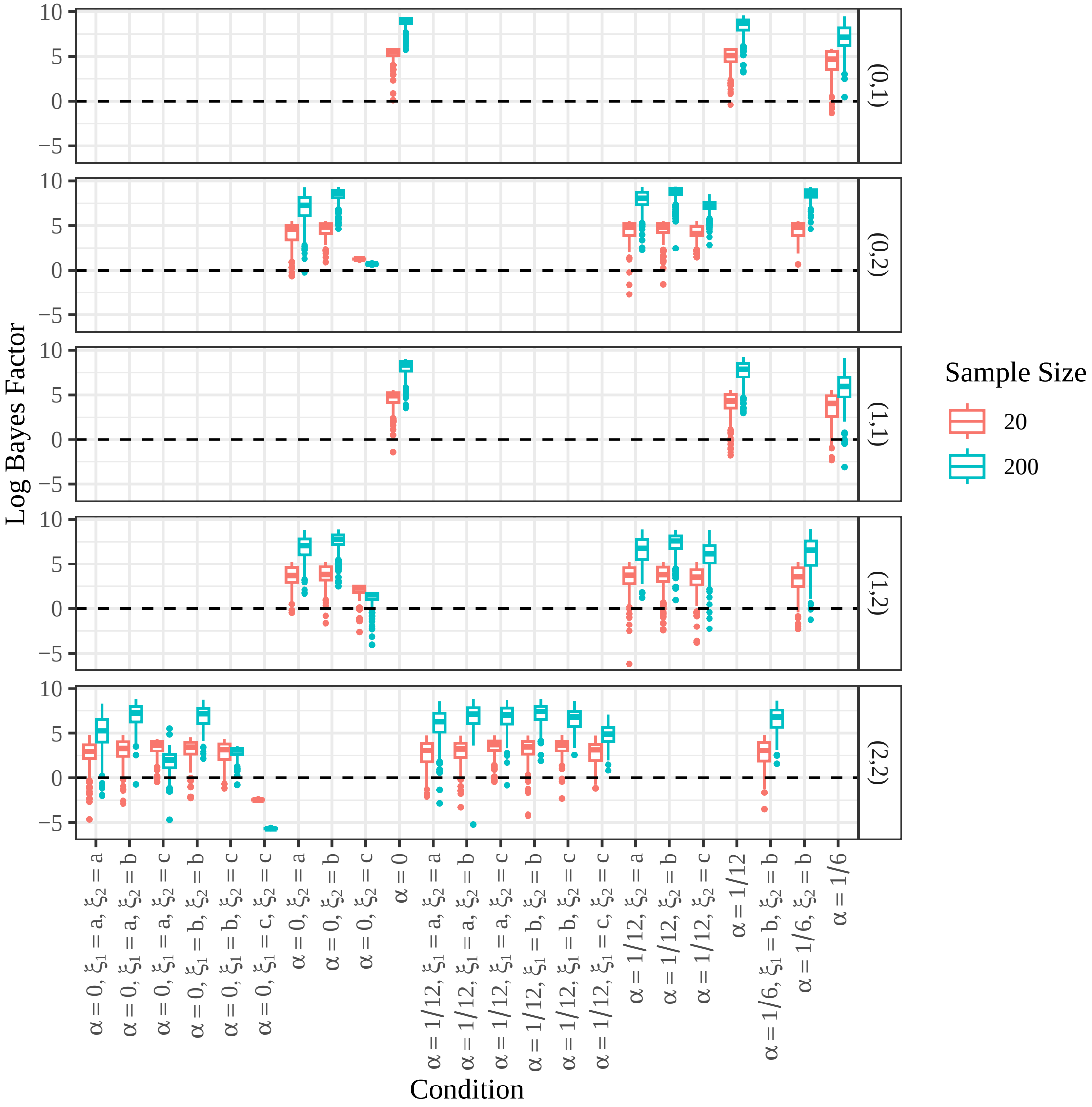

Figure S7: Box plots of the log Bayes factors from the Bayes tests of Section 2.3, when using known genotypes, from the null simulations in Section 3.1. Since the null is true, the log Bayes factors should be mostly above 0 (horizontal black dashed line). Row-facets index parent genotypes (ℓ_1, ℓ_2), and color indexes sample size. The x-axis indexes different values of the double reduction rate, α, and the preferential pairing parameters of the two parents, ξ_1 and ξ_2. A preferential pairing value of "a" indicates the lower bound in (S42), a value of "b" indicates 1/3, and a value of "c" indicates the upper bound in (S42). Preferential pairing only affects offspring genotype frequencies when the parent genotype is 2, and so an omission of ξ_1 or ξ_2 from the x-axis labels indicates a scenario where the corresponding parent genotype is not 2.

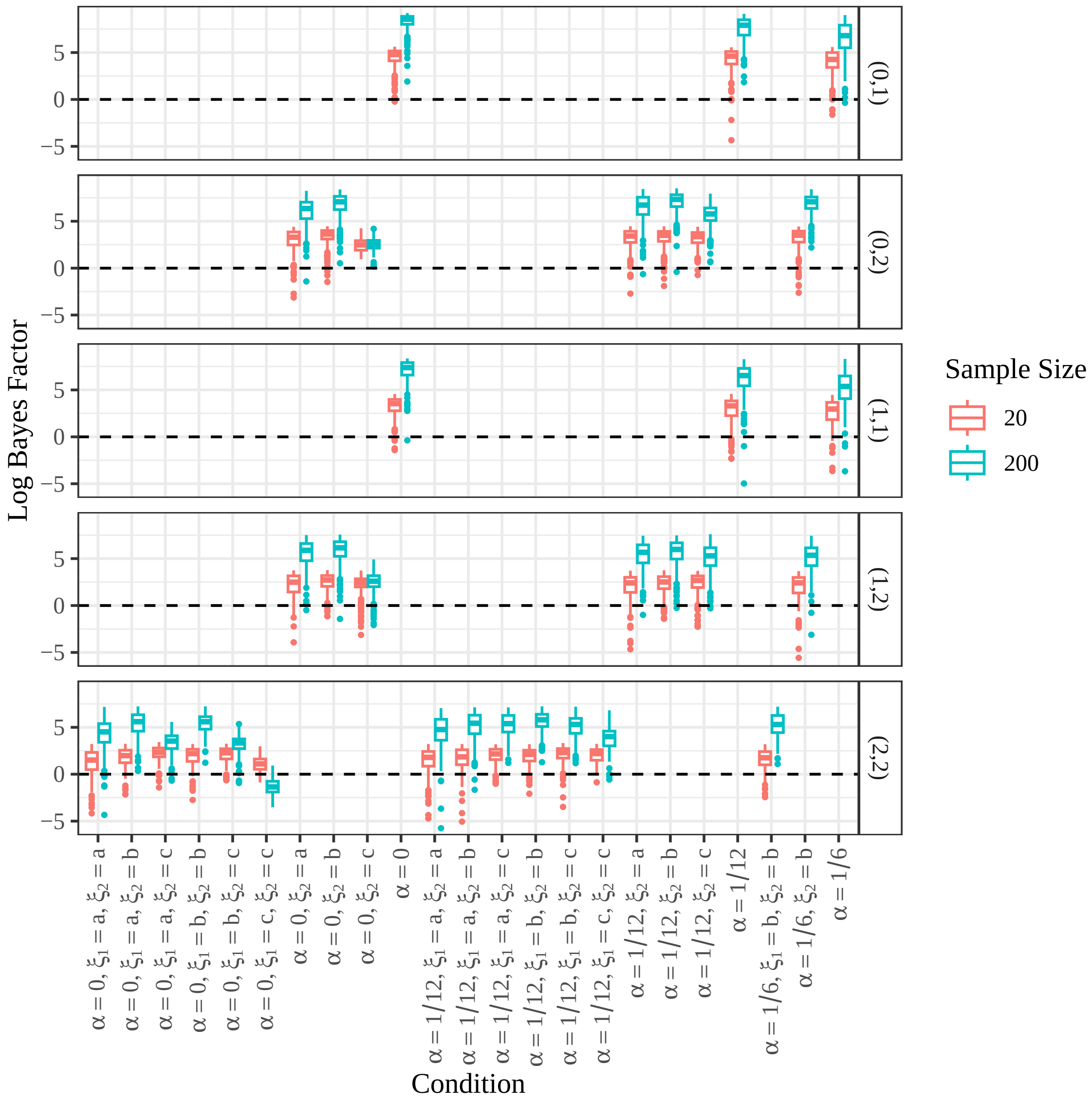

Figure S8: Box plots of the log Bayes factors from the Bayes tests of Section 2.3, using genotype likelihoods derived from read-counts simulated at a read-depth of 10, from the null simulations in Section 3.1. Since the null is true, the log Bayes factors should be mostly above 0 (horizontal black dashed line). Row-facets index parent genotypes (ℓ_1, ℓ_2), and color indexes sample size. The x-axis indexes different values of the double reduction rate, α, and the preferential pairing parameters of the two parents, ξ_1 and ξ_2. A preferential pairing value of "a" indicates the lower bound in (S42), a value of "b" indicates 1/3, and a value of "c" indicates the upper bound in (S42). Preferential pairing only affects offspring genotype frequencies when the parent genotype is 2, and so an omission of ξ_1 or ξ_2 from the x-axis labels indicates a scenario where the corresponding parent genotype is not 2.

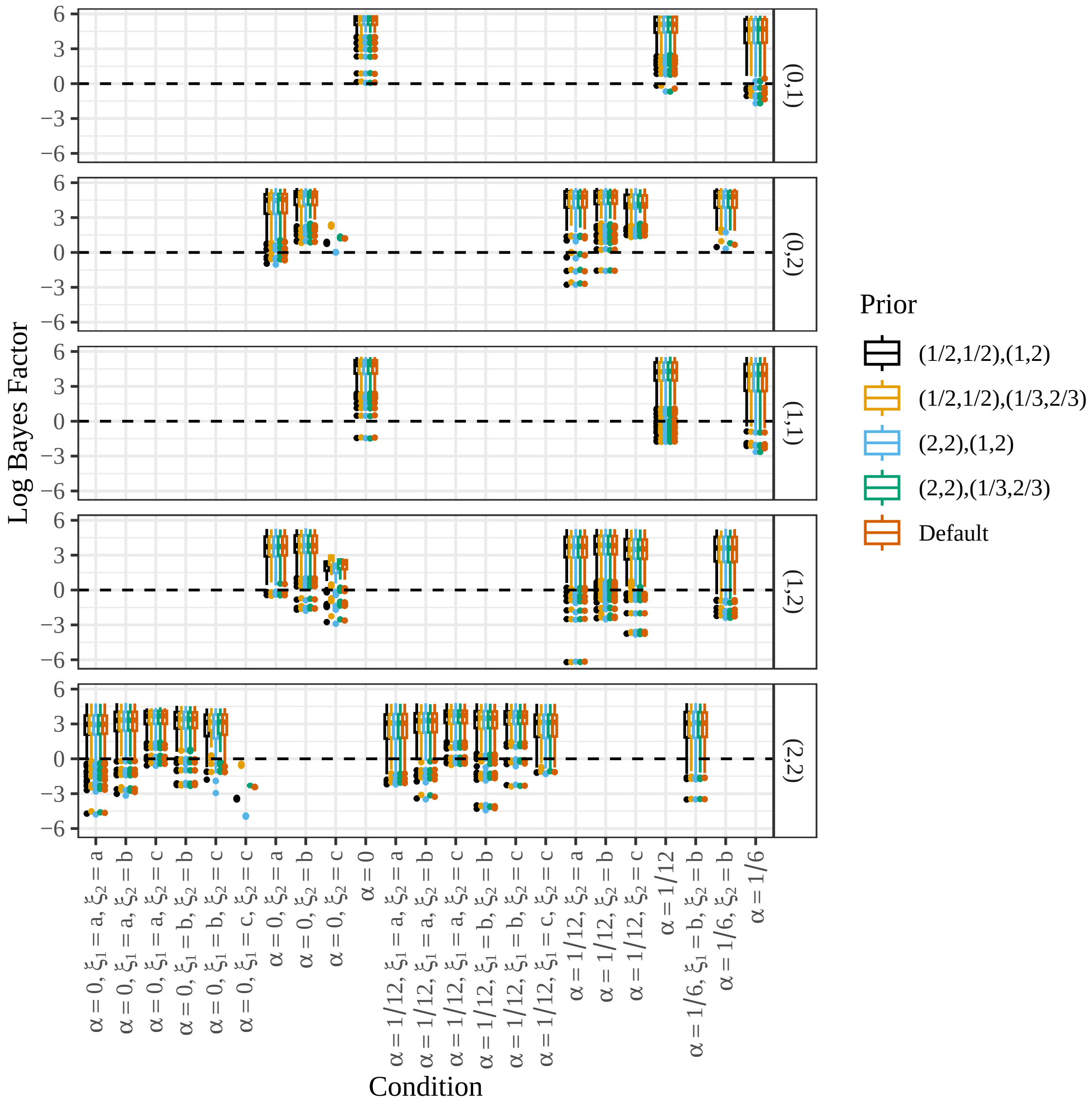

Figure S9: Box plots of the log Bayes factors under different priors (color) from the Bayes tests of Section 2.3, when using known genotypes and $n = 20$, from the null simulations in Section 3.1. In the color legend, the first pair of numbers are the shape parameters for the beta prior of τ, the second pair of numbers are the shape parameters for the beta priors for γ_1 and γ_2. Since the null is true, the log Bayes factors should be mostly above 0 (horizontal black dashed line). The log Bayes factors are mostly robust to prior choice. Row-facets index parent genotypes (ℓ_1, ℓ_2). The x-axis indexes different values of the double reduction rate, α, and the preferential pairing parameters of the two parents, ξ_1 and ξ_2. A preferential pairing value of "a" indicates the lower bound in (S42), a value of "b" indicates 1/3, and a value of "c" indicates the upper bound in (S42). Preferential pairing only affects offspring genotype frequencies when the parent genotype is 2, and so an omission of ξ_1 or ξ_2 from the x-axis labels indicates a scenario where the corresponding parent genotype is not 2.

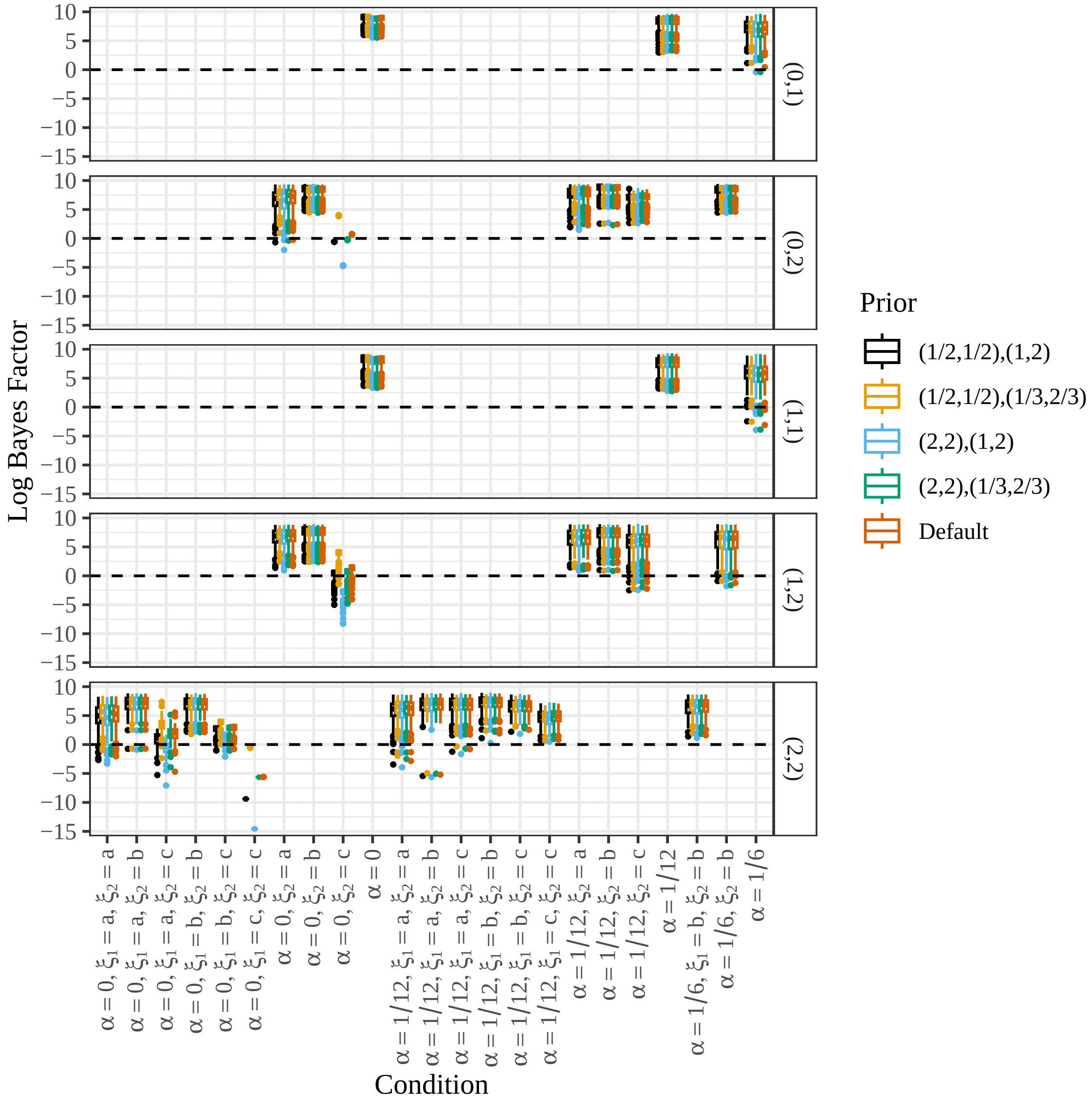

Figure S10: Box plots of the log Bayes factors under different priors (color) from the Bayes tests of Section 2.3, when using known genotypes and $n = 200$, from the null simulations in Section 3.1. In the color legend, the first pair of numbers are the shape parameters for the beta prior of τ, the second pair of numbers are the shape parameters for the beta priors for γ_1 and γ_2. Since the null is true, the log Bayes factors should be mostly above 0 (horizontal black dashed line). The log Bayes factors are mostly robust to prior choice. Row-facets index parent genotypes (ℓ_1, ℓ_2). The x-axis indexes different values of the double reduction rate, α, and the preferential pairing parameters of the two parents, ξ_1 and ξ_2. A preferential pairing value of "a" indicates the lower bound in (S42), a value of "b" indicates 1/3, and a value of "c" indicates the upper bound in (S42). Preferential pairing only affects offspring genotype frequencies when the parent genotype is 2, and so an omission of ξ_1 or ξ_2 from the x-axis labels indicates a scenario where the corresponding parent genotype is not 2.

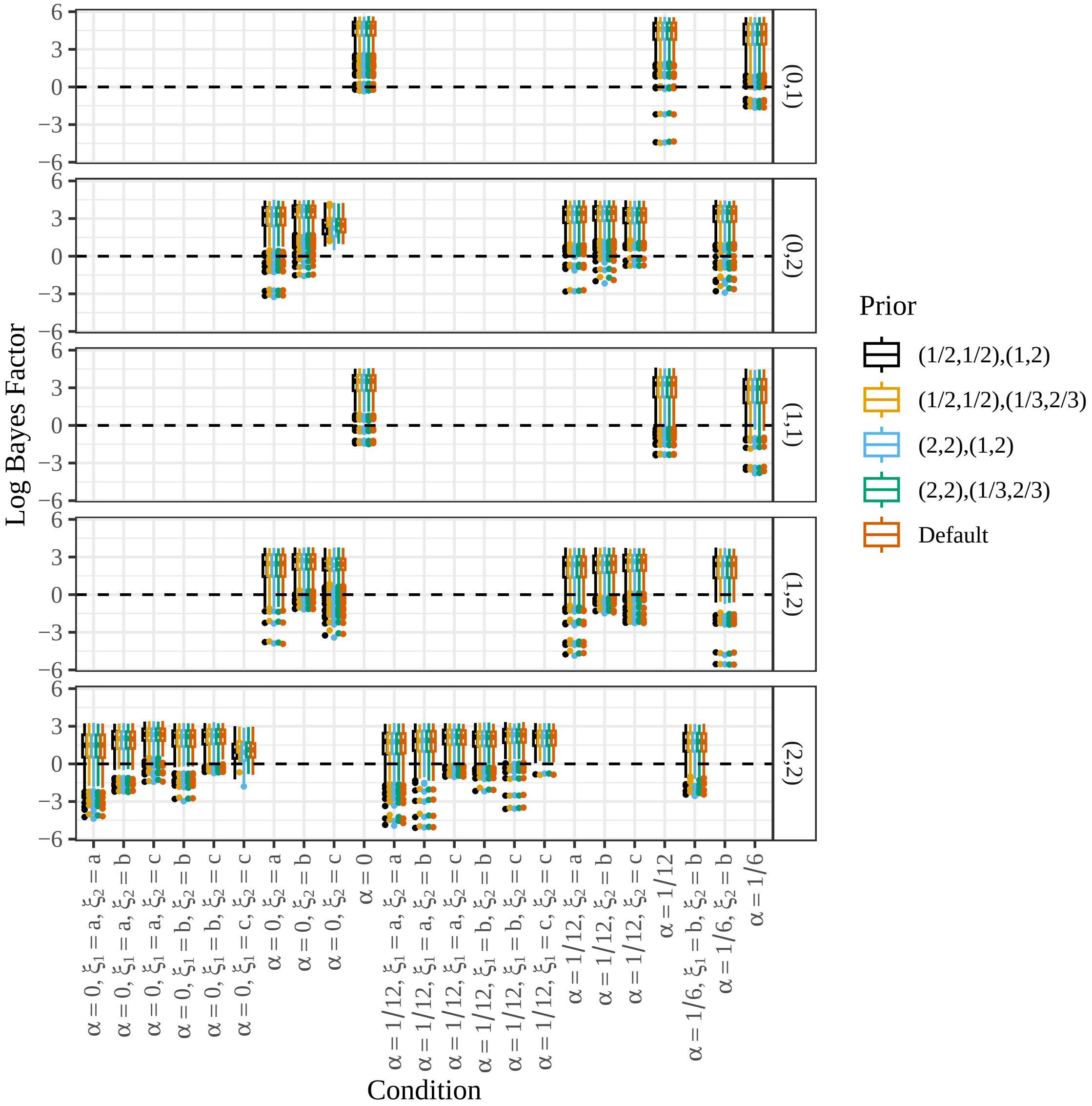

Figure S11: Box plots of the log Bayes factors under different priors (color) from the Bayes tests of Section 2.3, using genotype likelihoods derived from read-counts simulated at a read-depth of 10 and $n = 20$, from the null simulations in Section 3.1. In the color legend, the first pair of numbers are the shape parameters for the beta prior of τ, the second pair of numbers are the shape parameters for the beta priors for γ_1 and γ_2. Since the null is true, the log Bayes factors should be mostly above 0 (horizontal black dashed line). The log Bayes factors are mostly robust to prior choice. Row-facets index parent genotypes (ℓ_1, ℓ_2). The x-axis indexes different values of the double reduction rate, α, and the preferential pairing parameters of the two parents, ξ_1 and ξ_2. A preferential pairing value of "a" indicates the lower bound in (S42), a value of "b" indicates 1/3, and a value of "c" indicates the upper bound in (S42). Preferential pairing only affects offspring genotype frequencies when the parent genotype is 2, and so an omission of ξ_1 or ξ_2 from the x-axis labels indicates a scenario where the corresponding parent genotype is not 2.

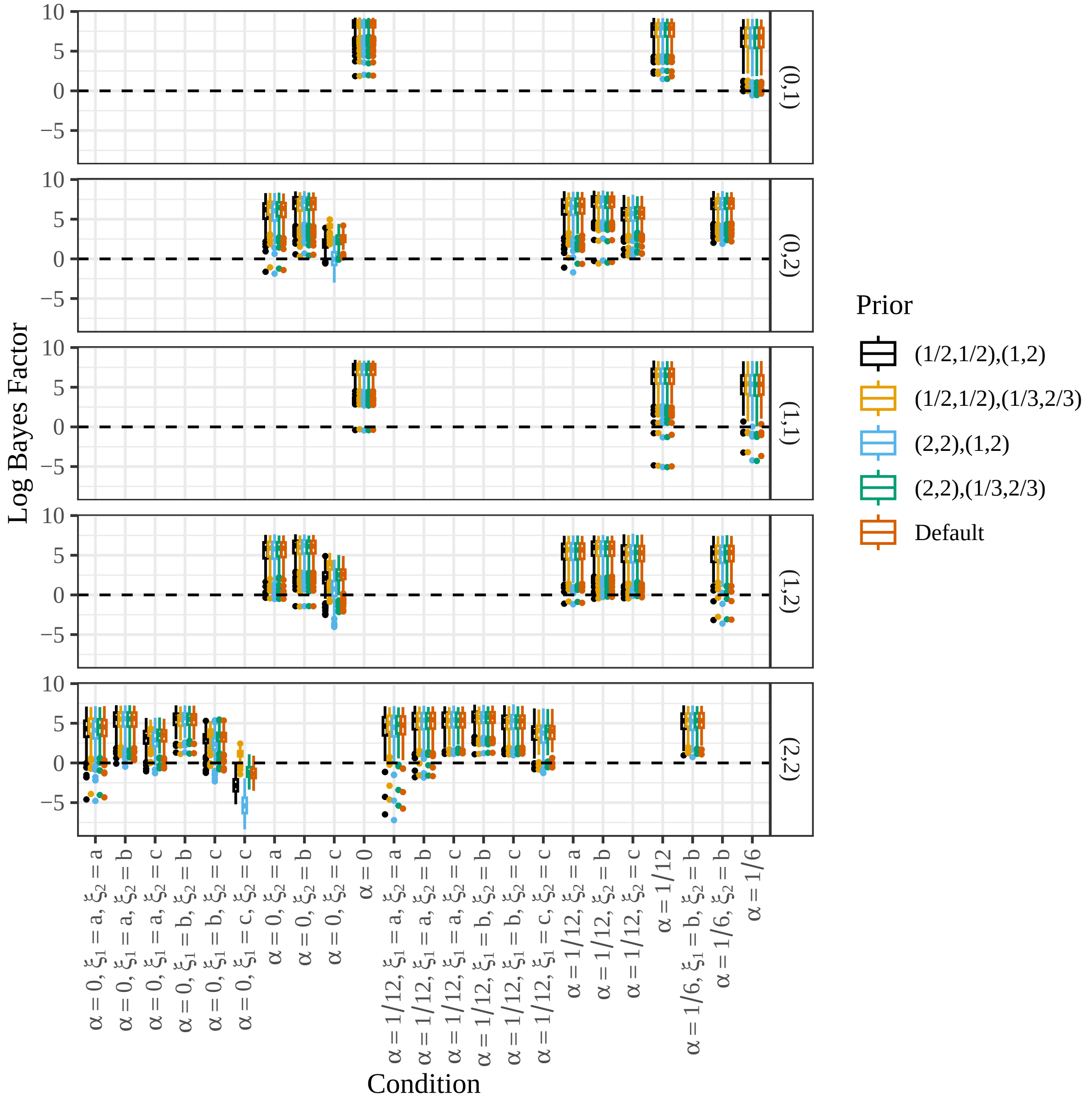

Figure S12: Box plots of the log Bayes factors under different priors (color) from the Bayes tests of Section 2.3, using genotype likelihoods derived from read-counts simulated at a read-depth of 10 and $n = 200$, from the null simulations in Section 3.1. In the color legend, the first pair of numbers are the shape parameters for the beta prior of τ, the second pair of numbers are the shape parameters for the beta priors for γ_1 and γ_2. Since the null is true, the log Bayes factors should be mostly above 0 (horizontal black dashed line). The log Bayes factors are mostly robust to prior choice. Row-facets index parent genotypes (ℓ_1, ℓ_2). The x-axis indexes different values of the double reduction rate, α, and the preferential pairing parameters of the two parents, ξ_1 and ξ_2. A preferential pairing value of “a” indicates the lower bound in (S42), a value of “b” indicates 1/3, and a value of “c” indicates the upper bound in (S42). Preferential pairing only affects offspring genotype frequencies when the parent genotype is 2, and so an omission of ξ_1 or ξ_2 from the x-axis labels indicates a scenario where the corresponding parent genotype is not 2.

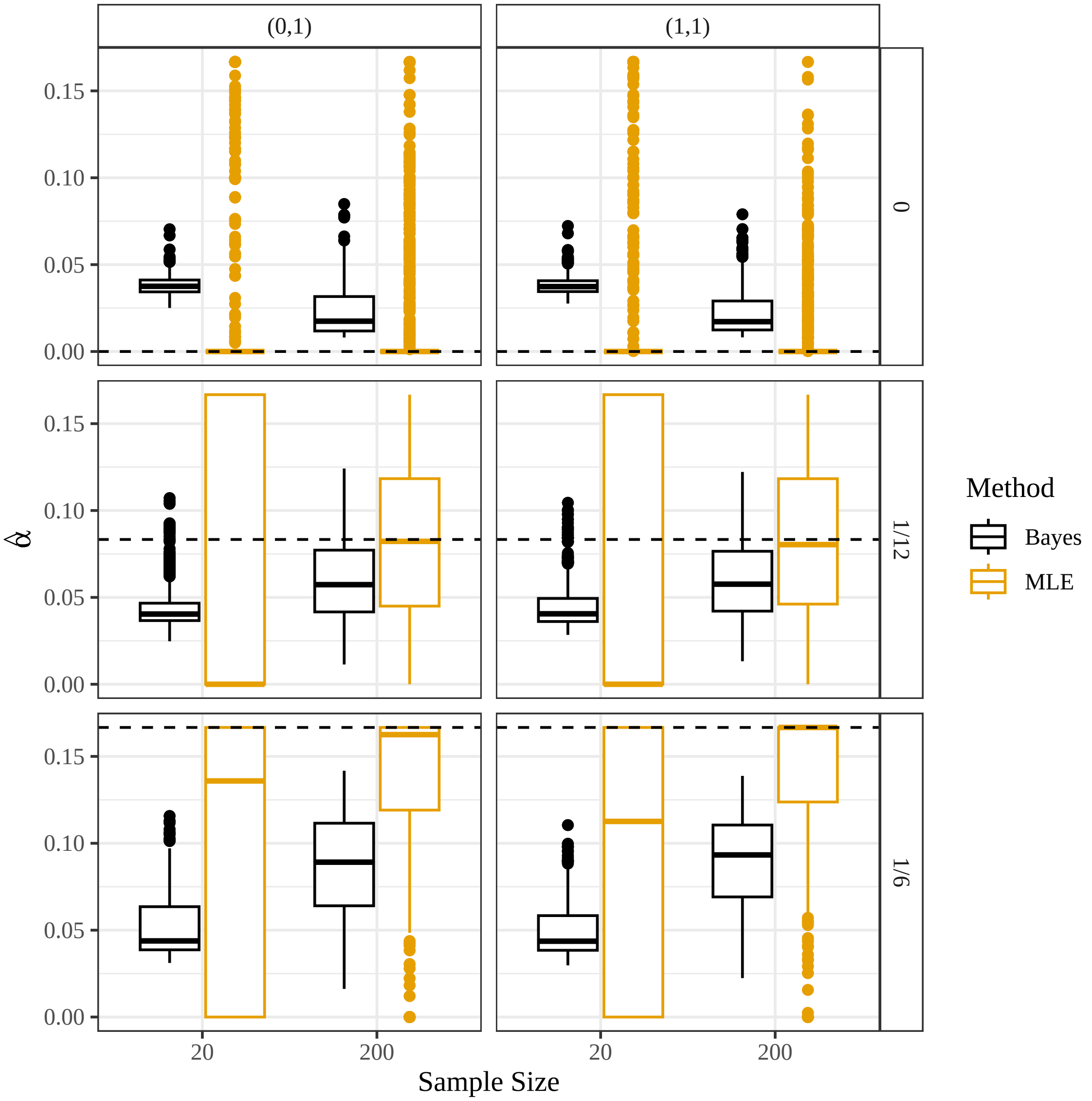

Figure S13: Box plots of estimates of the double reduction rate $\hat{\alpha}$ (y-axis) stratified by sample size (x-axis), parent genotypes (column facets), true double reduction rate (row facets and horizontal dashed lines), and estimation method (Bayesian posterior mean or maximum likelihood). Estimates are biased and have high variance, indicating that they are unreliable, even for large sample sizes.

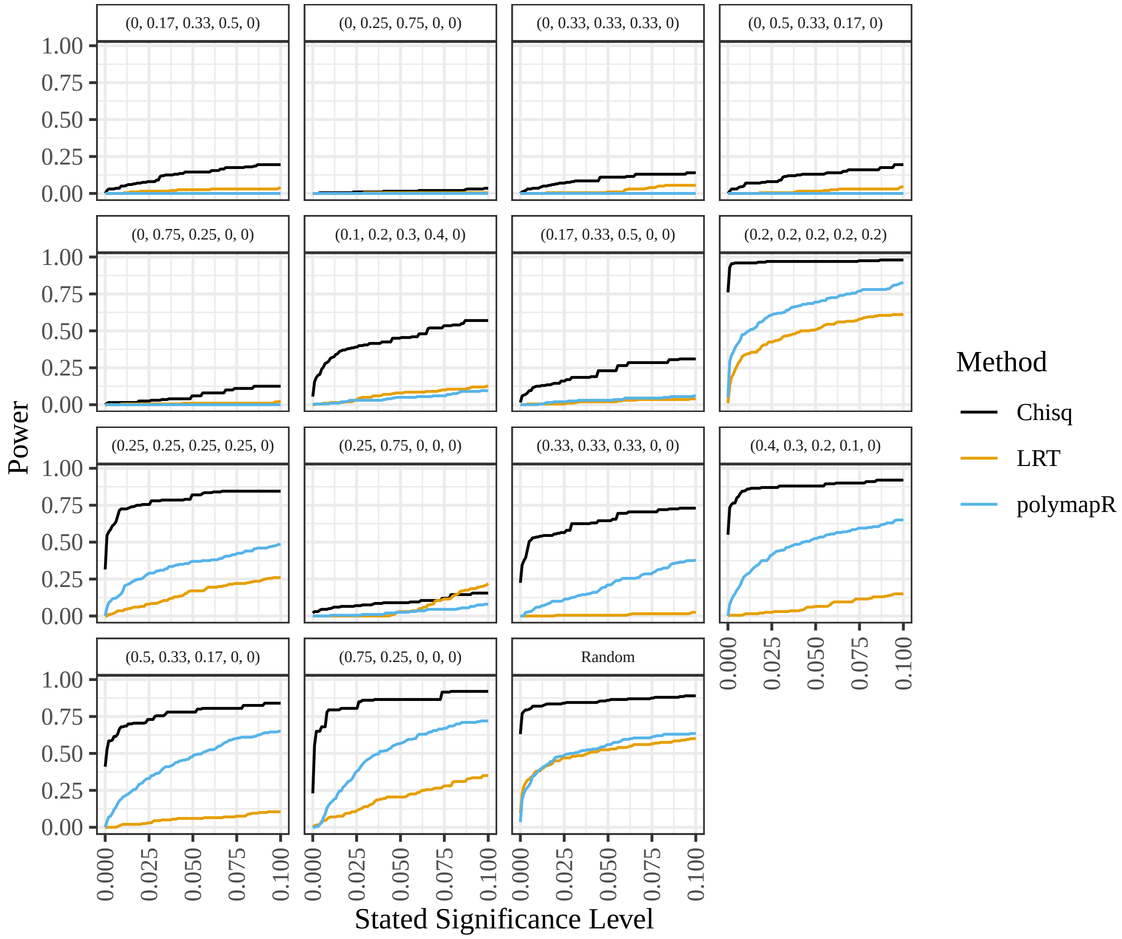

Figure S14: Power (y-axis) of different alternatives (facets) for different methods (color) at realistic levels of stated Type I error control (x-axis). This is for the scenario at a read-depth of 10 and a sample size of 20. Only the likelihood ratio test actually controls Type I error at the stated level, and so this plot demonstrates the unavoidable loss in power caused by correctly controlling Type I error.

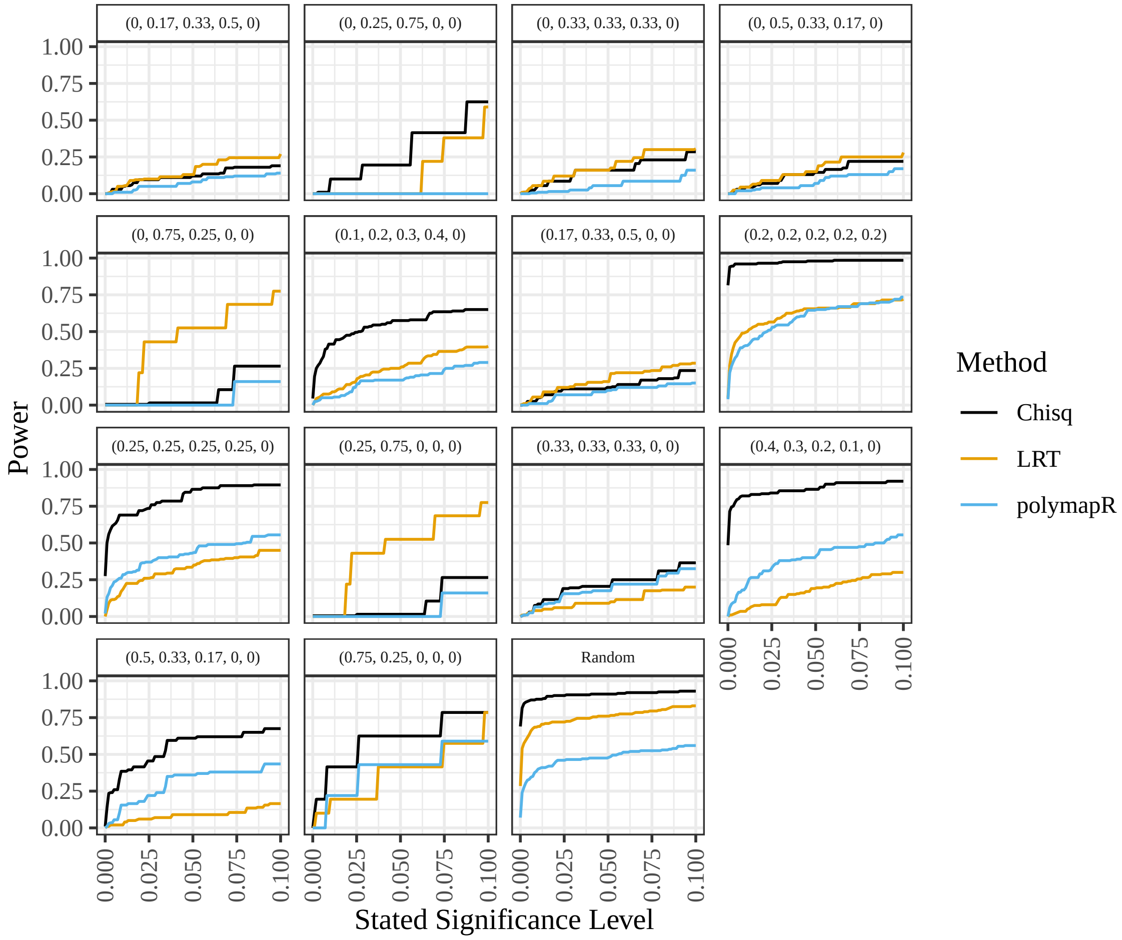

Figure S15: Power (y-axis) of different alternatives (facets) for different methods (color) at realistic levels of stated Type I error control (x-axis). This is for the scenario at a read-depth of infinity and a sample size of 20. Only the likelihood ratio test actually controls Type I error at the stated level, and so this plot demonstrates the (typically) unavoidable loss in power caused by correctly controlling Type I error.

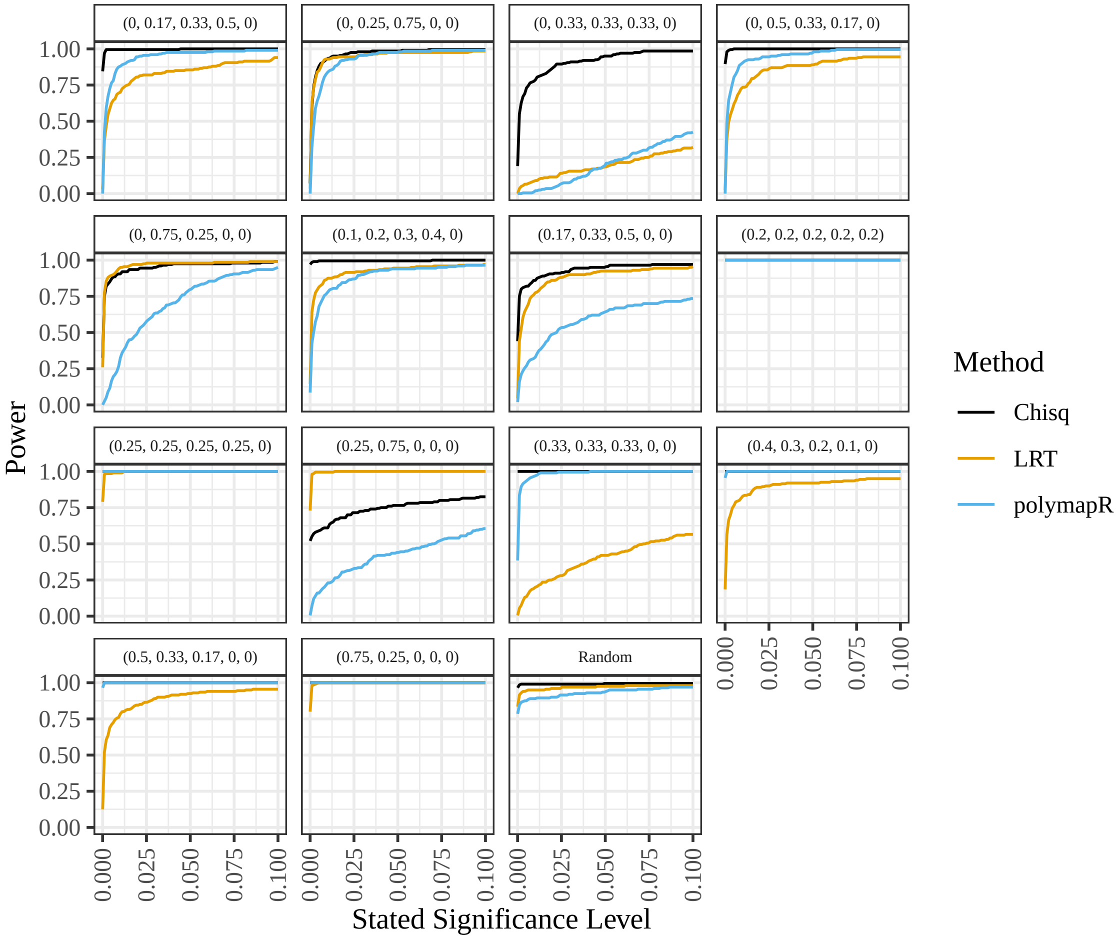

Figure S16: Power (y-axis) of different alternatives (facets) for different methods (color) at realistic levels of stated Type I error control (x-axis). This is for the scenario at a read-depth of 10 and a sample size of 200. Only the likelihood ratio test actually controls Type I error at the stated level, and so this plot demonstrates the (typically) unavoidable loss in power caused by correctly controlling Type I error.

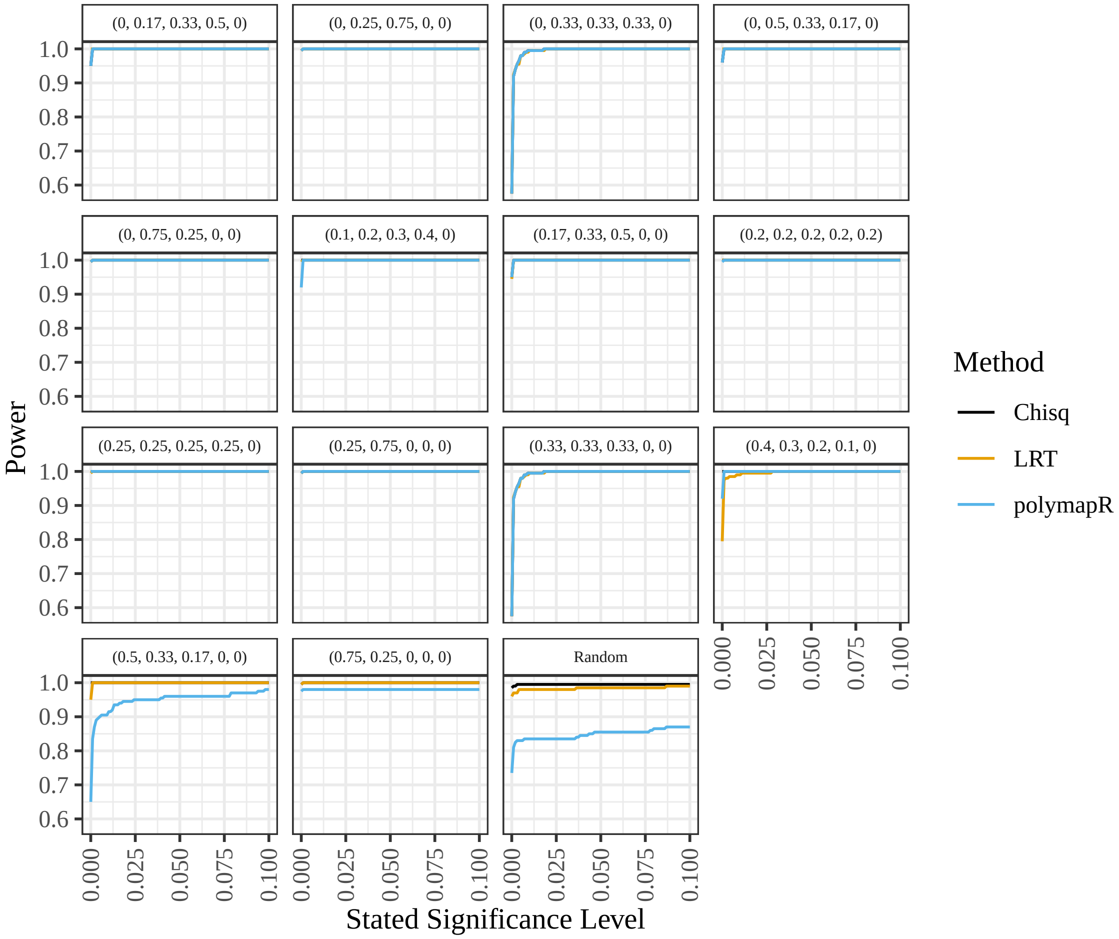

Figure S17: Power (y-axis) of different alternatives (facets) for different methods (color) at realistic levels of stated Type I error control (x-axis). This is for the scenario at a read-depth of infinity and a sample size of 200. Only the likelihood ratio test actually controls Type I error at the stated level, and so this plot demonstrates the (typically) unavoidable loss in power caused by correctly controlling Type I error.

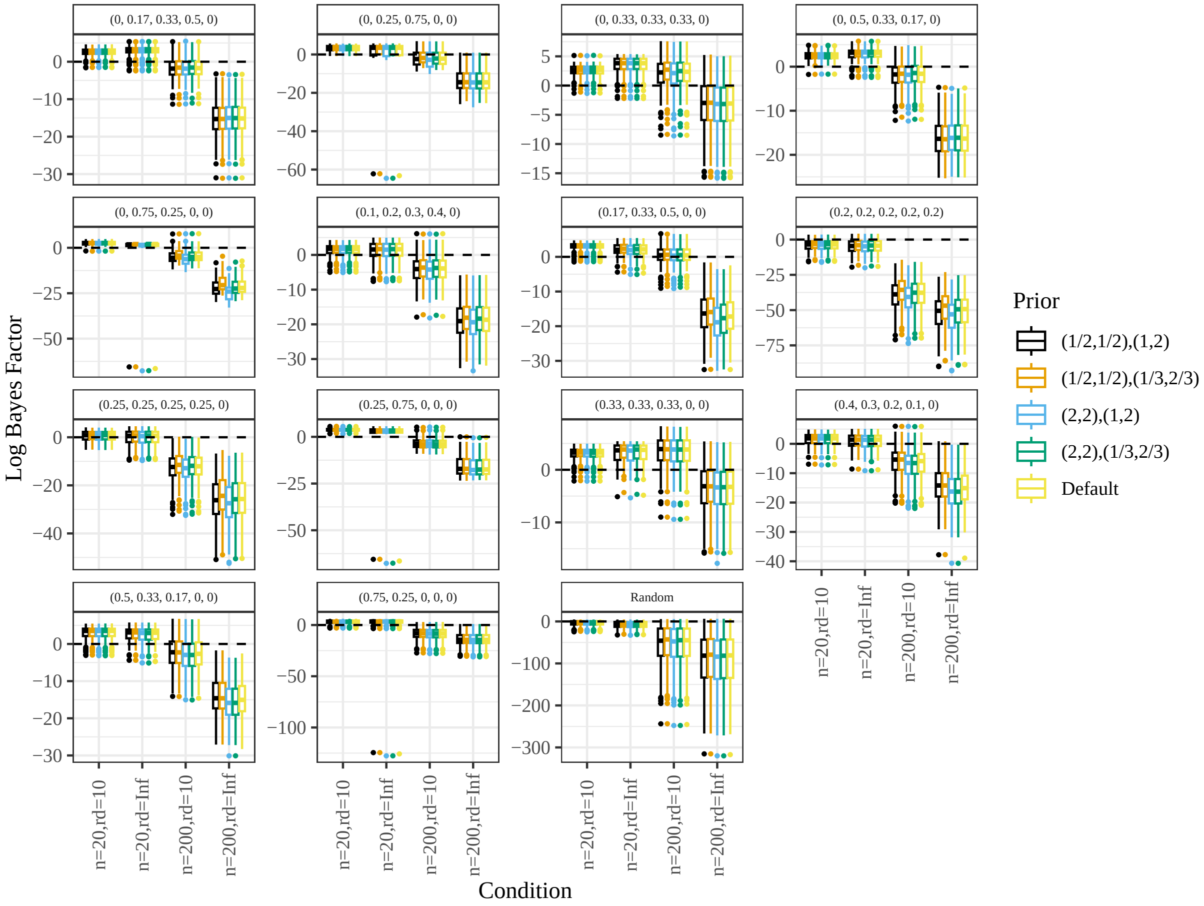

Figure S18: Box plots of the log Bayes factors (y-axis) from the Bayesian method of Section 2.3 under different priors (color), at different sample sizes (n), different read-depths (rd), and different alternative scenarios (facets). In the color legend, the first pair of numbers are the shape parameters for the beta prior of τ, the second pair of numbers are the shape parameters for the beta priors for γ_1 and γ_2. Negative values indicate support for the alternative, and these simulations were run when the alternative was true (Section 3.2), and so more negative values indicate superior performance. Our Bayes test provides negative values for most scenarios.

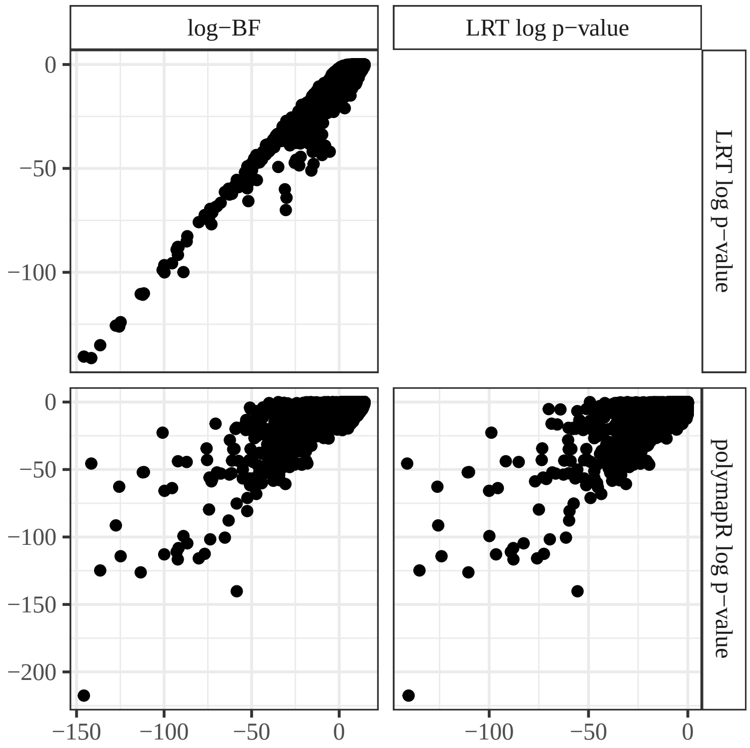

Figure S19: Pairs plot of the log Bayes factors from the Bayes test from Section 2.3, and the log p-values from the LRT of Section 2.2 and the polymapR test of Section 1.1. The Bayes test and LRT provide more concordant results.

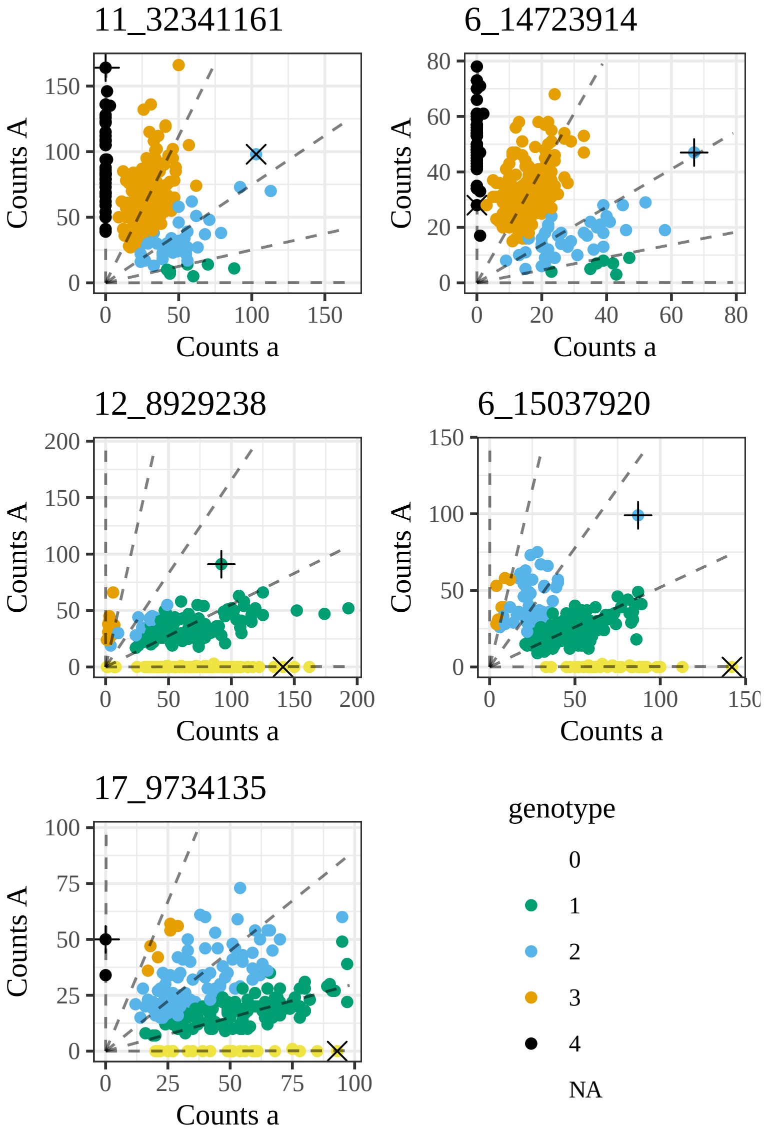

Figure S20: Genotype plots [Gerard et al., 2018] of five SNPs (titles) where the LRT indicates strong segregation distortion, but polymapR indicates no segregation distortion. The p-values and information on these SNPs are provided in Table S1. The x-axis contains the counts of the alternative allele, and the y-axis contains the counts of the reference allele. The dashed lines radiating from the origin are the expected counts under the fitted model of updog. The colors indicate posterior mode genotype. The "+" and "×" symbols indicate the parents.

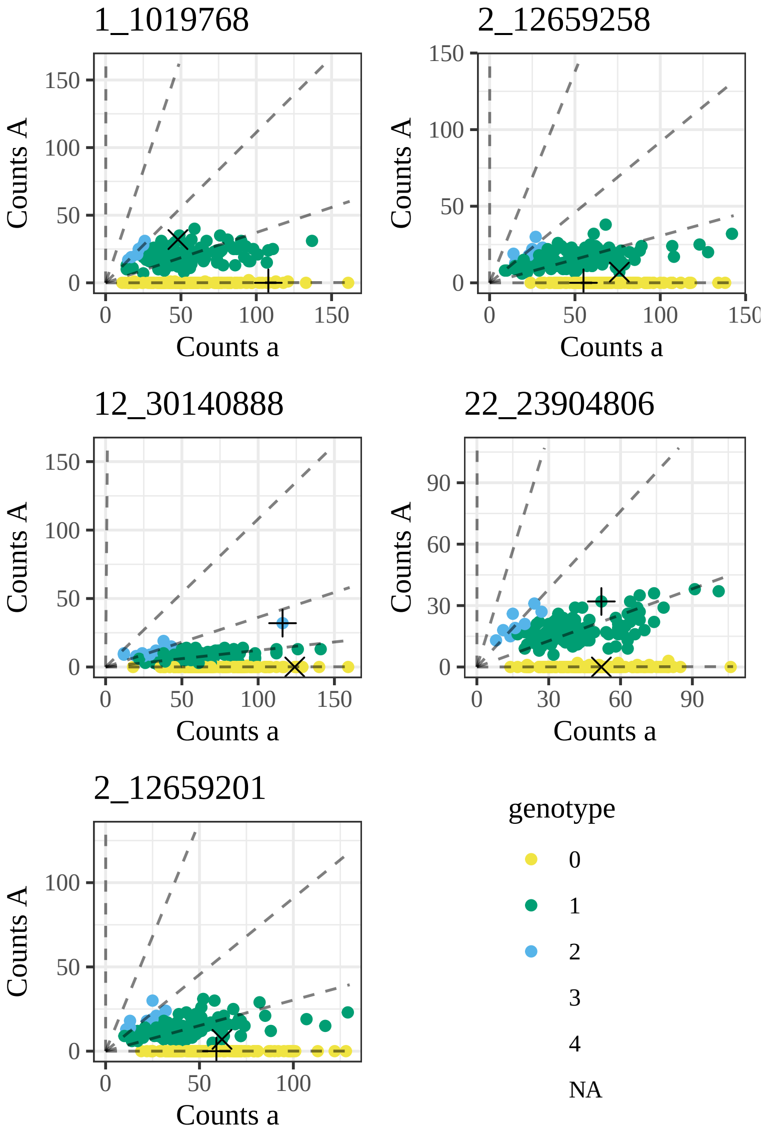

Figure S21: Genotype plots [Gerard et al., 2018] of five SNPs (titles) where the LRT indicates no segregation distortion, but polymapR indicates strong segregation distortion. The p-values and information on these SNPs are provided in Table S1. The x-axis contains the counts of the alternative allele, and the y-axis contains the counts of the reference allele. The dashed lines radiating from the origin are the expected counts under the fitted model of updog. The colors indicate posterior mode genotype. The "+" and "×" symbols indicate the parents.

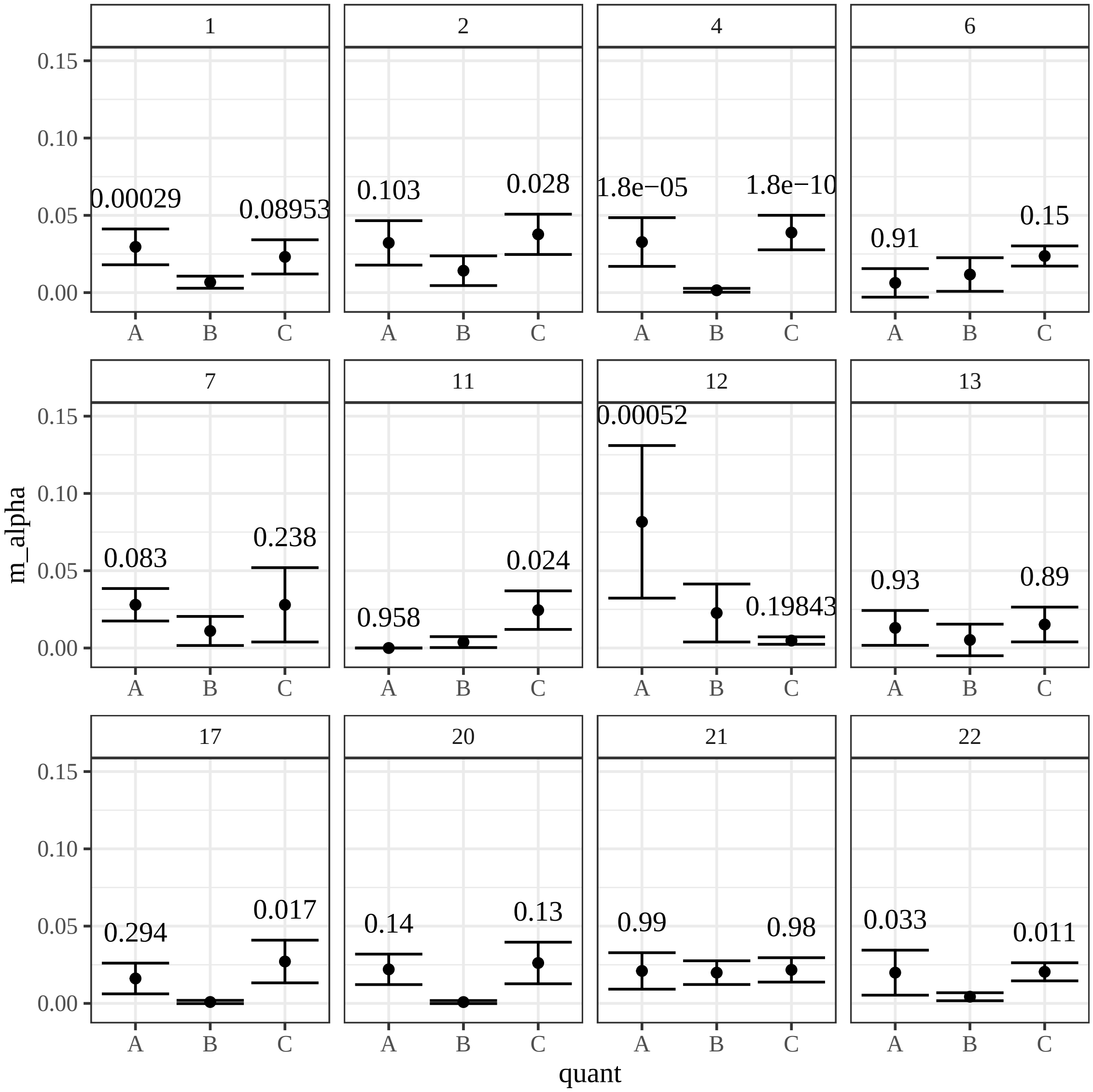

Figure S22: Average (dots) and plus or minus two standard errors (error bars) for the mean double reduction rate estimates for the first 10% of SNPs ("A"), middle 20% of SNPs ("B"), and last 10% of SNPs ("C") for the 12 linkage groups from the blueberry data (Facets) at SNPs where parent 1 was simplex and parent 2 was nullplex. The numbers above the "A" and "C" error bars are the Tukey adjusted p-values [Tukey, 1949] comparing groups "A"-"B" and "B"-"C", respectively.

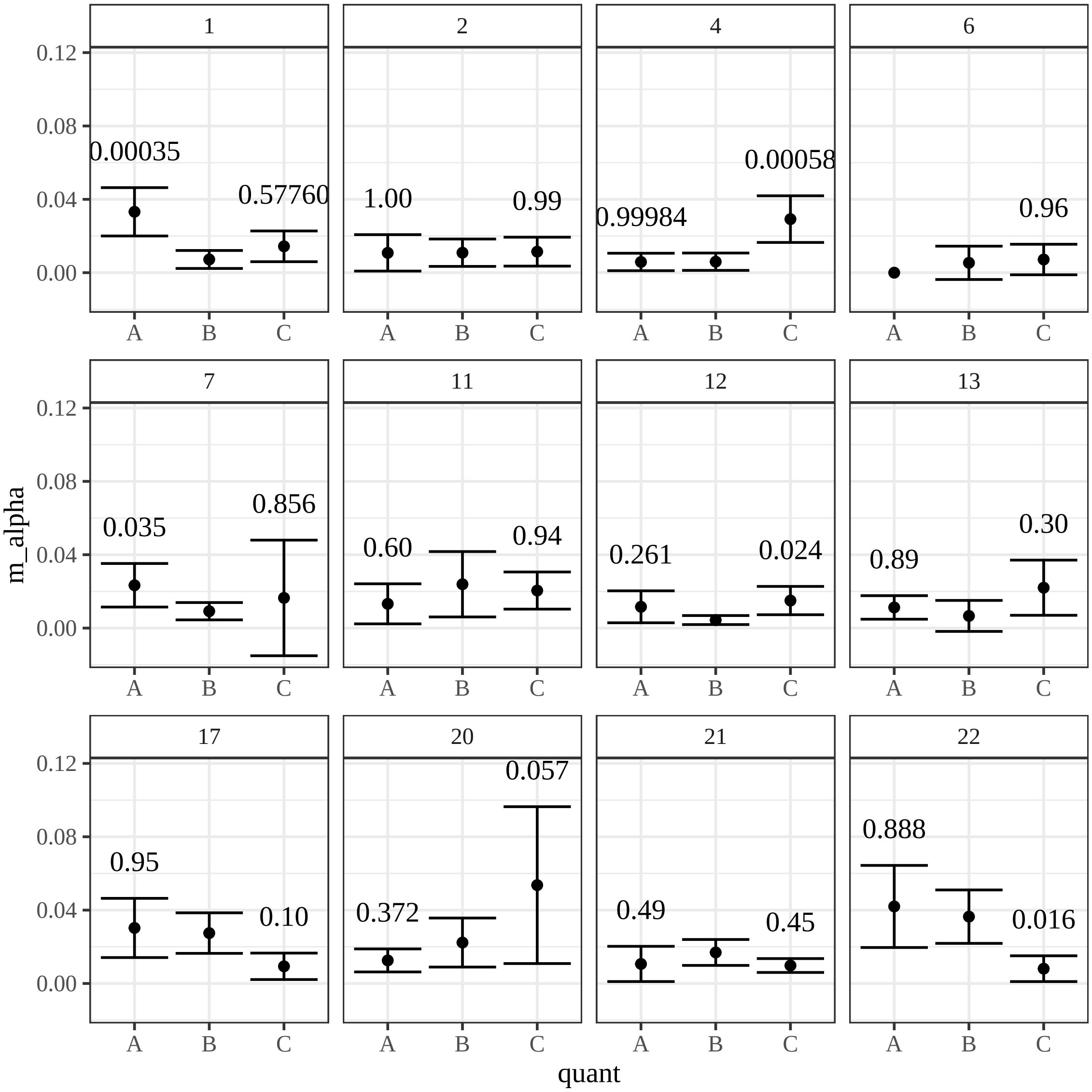

Figure S23: Average (dots) and plus or minus two standard errors (error bars) for the mean double reduction rate estimates for the first 10% of SNPs ("A"), middle 20% of SNPs ("B"), and last 10% of SNPs ("C") for the 12 linkage groups from the blueberry data (Facets) at SNPs where parent 1 was nullplex and parent 2 was simplex. The numbers above the "A" and "C" error bars are the Tukey adjusted p-values [Tukey, 1949] comparing groups "A"-"B" and "B"-"C", respectively.

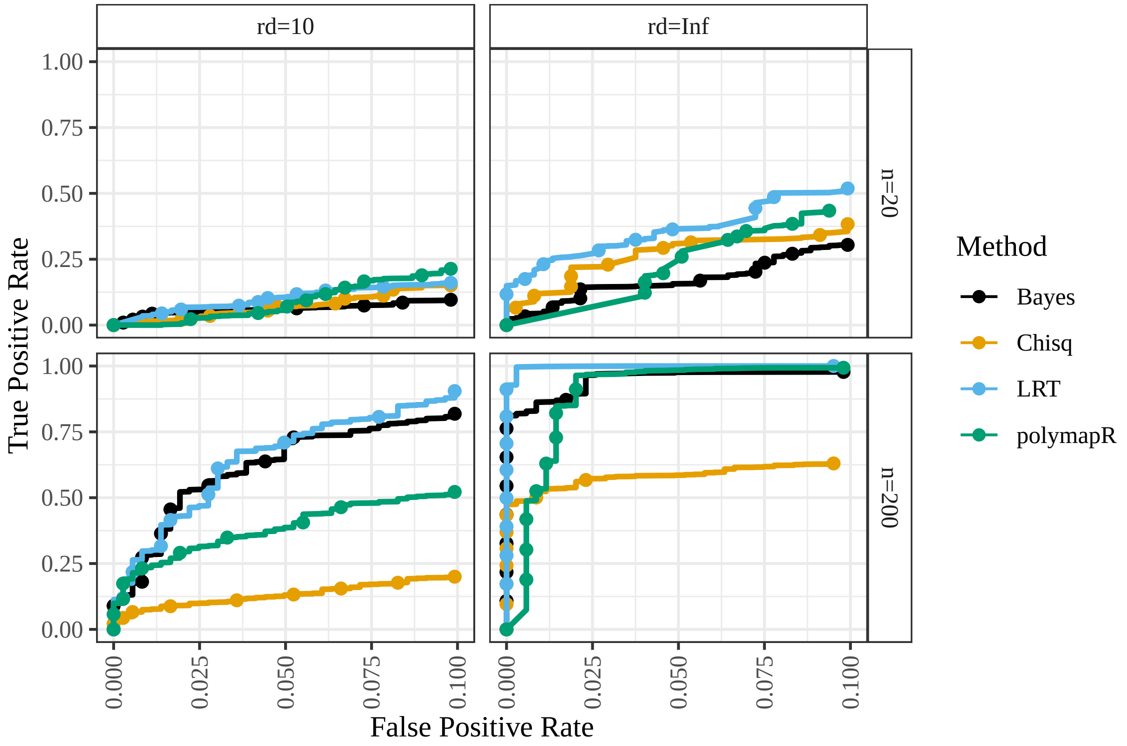

Figure S24: ROC curve at realistic levels of Type I error rate. The Type I error rate (false positive rate) on the x-axis is plotted against power (true positive rate) on the y-axis for various methods (color) using all 9000 null simulation scenarios and 375 alternative scenarios, randomly sampled from the 3000 alternative scenarios in Figure 2, per ROC curve. The alternative scenarios represent 4% of all cases, consistent with the blueberry data in Section 3.3. This is a general overview for the simulation performance of the various methods. The likelihood ratio test is generally the best performer, and the Bayes test is second best for large sample sizes.

References

P. M. Bourke, R. E. Voorrips, R. G. F. Visser, and C. Maliepaard. The double-reduction landscape in tetraploid potato as revealed by a high-density linkage map. *Genetics*, 201(3):853–863, 2015. doi: 10.1534/genetics.115.181008.

P. M. Bourke, P. Arens, R. E. Voorrips, G. D. Esselink, C. F. S. Koning-Boucoiran, W. P. C. van't Westende, T. Santos Leonardo, P. Wissink, C. Zheng, G. Geest, R. G. F. Visser, F. A. Krens, M. J. M. Smulders, and C. Maliepaard. Partial preferential chromosome pairing is genotype dependent in tetraploid rose. *The Plant Journal*, 90(2):330–343, 2017. doi: 10.1111/tpj.13496.

P. M. Bourke, G. van Geest, R. E. Voorrips, J. Jansen, T. Kranenburg, A. Shahin, R. G. F. Visser, P. Arens, M. J. M. Smulders, and C. Maliepaard. polymapR—linkage analysis and genetic map construction from F1 populations of outcrossing polyploids. *Bioinformatics*, 34(20):3496–3502, 2018. doi: 10.1093/bioinformatics/bty371.

D. Cao, T. C. Osborn, and R. W. Doerge. Correct estimation of preferential chromosome pairing in autotetraploids. *Genome Research*, 14(3):459–462, 2004. doi: 10.1101/gr.1596604.

A. Cornille, A. Salcedo, D. Kryvokhyzha, S. Glémin, K. Holm, S. Wright, and M. Lascoux. Genomic signature of successful colonization of Eurasia by the allopolyploid shepherd's purse (*capsella bursa-pastoris*). *Molecular ecology*, 25(2):616–629, 2016. doi: 10.1111/mec.13491.

R. A. Fisher and K. Mather. The inheritance of style length in *Lythrum salicaria*. *Annals of Eugenics*, 12(1):1–23, 1943. doi: 10.1111/j.1469-1809.1943.tb02307.x.

R. Fjellstrom, P. Beuselinck, and J. Steiner. RFLP marker analysis supports tetrasonic inheritance in *Lotus corniculatus L.* *Theoretical and Applied Genetics*, 102(5):718–725, 2001. doi: 10.1007/s001220051702.

D. Gerard. Comment on three papers about Hardy–Weinberg equilibrium tests in autopolyploids. *Frontiers in Genetics*, 13, 2022a. doi: 10.3389/fgene.2022.1027209.

D. Gerard. Double Reduction Estimation and Equilibrium Tests in Natural Autopolyploid Populations. *Biometrics*, 79(3):2143–2156, 2022b. doi: 10.1111/biom.13722.

D. Gerard and L. F. V. Ferrão. Priors for genotyping polyploids. *Bioinformatics*, 36(6):1795–1800, 2019. doi: 10.1093/bioinformatics/btz852.

D. Gerard, L. F. V. Ferrão, A. A. F. Garcia, and M. Stephens. Genotyping polyploids from messy sequencing data. *Genetics*, 210(3):789–807, 2018. doi: 10.1534/genetics.118.301468.

K. Haynes and D. Douches. Estimation of the coefficient of double reduction in the cultivated tetraploid potato. *Theoretical and Applied Genetics*, 85(6-7):857–862, 1993. doi: 10.1007/BF00225029.

K. Huang, T. Wang, D. W. Dunn, P. Zhang, X. Cao, R. Liu, and B. Li. Genotypic frequencies at equilibrium for polysomic inheritance under double-reduction. *G3: Genes |Genomes |Genetics*, 9(5):1693–1706, 2019. doi: 10.1534/g3.119.400132.

M. Mollinari, B. A. Olukolu, G. d. S. Pereira, A. Khan, D. Gemenet, G. C. Yencho, and Z.-B. Zeng. Unraveling the hexaploid sweetpotato inheritance using ultra-dense multilocus mapping. *G3: Genes, Genomes, Genetics*, 10(1):281–292, 2020. doi: 10.1534/g3.119.400620.

M. S. Olson. Bayesian procedures for discriminating among hypotheses with discrete distributions: Inheritance in the tetraploid *Astilbe biternata*. *Genetics*, 147(4):1933–1942, 1997. URL `https://www.genetics.org/content/147/4/1933`.

L. Qu and J. Hancock. Detecting and mapping repulsion-phase linkage in polyploids with polysomic inheritance. *Theoretical and Applied Genetics*, 103(1):136–143, 2001. doi: 10.1007/s001220100647.

M. Stift, C. Berenos, P. Kuperus, and P. H. van Tienderen. Segregation models for disomic, tetrasomic and intermediate inheritance in tetraploids: a general procedure applied to *Rorippa* (yellow cress) microsatellite data. *Genetics*, 179(4):2113–2123, 2008. doi: 10.1534/genetics.107.085027.

R. Sun. Estimating preferential pairing in polyploids. Master's thesis, American University, 2020. Advisor: David Gerard.

M. Swaminathan and H. Howard. The cytology and genetics of the potato (*Solanum tuberosum*) and related species. *Bibliographia genet.*, 16:1–192, 1953.

G. Tai. Estimation of double reduction and genetic parameters in autotetraploids based on 4x–2x and 4x–4x matings. *Heredity*, 49(3):331–335, 1982a. doi: 10.1038/hdy.1982.106.

G. Tai. Estimation of double reduction and genetic parameters of autotetraploids. *Heredity*, 49(1): 63–70, 1982b. doi: 10.1038/hdy.1982.65.

J. W. Tukey. Comparing individual means in the analysis of variance. *Biometrics*, 5(2):99–114, 1949. doi: 10.2307/3001913.

R. Wu, M. Gallo-Meagher, R. C. Littell, and Z.-B. Zeng. A general polyploid model for analyzing gene segregation in outcrossing tetraploid species. *Genetics*, 159(2):869–882, 10 2001. doi: 10.1093/genetics/159.2.869.

C. Zheng, R. E. Voorrips, J. Jansen, C. A. Hackett, J. Ho, and M. C. Bink. Probabilistic multilocus haplotype reconstruction in outcrossing tetraploids. *Genetics*, 203(1):119–131, 2016. doi: 10.1534/genetics.115.185579.

C. Zheng, R. R. Amadeu, P. R. Munoz, and J. B. Endelman. Haplotype reconstruction in connected tetraploid F1 populations. *Genetics*, 219(2), 2021. doi: 10.1093/genetics/iyab106. iyab106.
